# CRISPR-Cas– induced IRF3 and MAVS knockouts in a salmonid cell line disrupt PRR signaling and affect viral replication

**DOI:** 10.3389/fimmu.2023.1214912

**Published:** 2023-07-17

**Authors:** Yorick A. van der Wal, Henriette Nordli, Allan Akandwanaho, Linn Greiner-Tollersrud, Jaap Kool, Jorunn B. Jørgensen

**Affiliations:** ^1^ Vaxxinova Research & Development GmbH, Münster, Germany; ^2^ Norwegian College of Fishery Science, Faculty of Biosciences, Fisheries & Economics, UiT The Arctic University of Norway, Tromsø, Norway

**Keywords:** Salmon alphavirus, CHSE-214, CRISPR-Cas, IFN responses, PRR signaling, MAVS, IRF, IPVN

## Abstract

**Background:**

Interferon (IFN) responses are critical in the resolution of viral infections and are actively targeted by many viruses. They also play a role in inducing protective responses after vaccination and have been successfully tested as vaccine adjuvants. IFN responses are well conserved and function very similar in teleosts and mammals. Like in mammals, IFN responses in piscine cells are initiated by intracellular detection of the viral infection by different pattern recognition receptors. Upon the recognition of viral components, IFN responses are rapidly induced to combat the infection. However, many viruses may still replicate and be able to inhibit or circumvent the IFN response by different means.

**Methods:**

By employing CRISPR Cas9 technology, we have disrupted proteins that are central for IFN signaling in the salmonid cell line CHSE-214. We successfully generated KO clones for the mitochondrial antiviral signaling protein MAVS, the transcription factors IRF3 and IRF7-1, as well as a double KO for IRF7-1/3 using an optimized protocol for delivery of CRISPR-Cas ribonucleoproteins through nucleofection.

**Results:**

We found that MAVS and IRF3 KOs inhibited IFN and IFN-stimulated gene induction after intracellular poly I:C stimulation as determined through gene expression and promoter activation assays. In contrast, the IRF7-1 KO had no clear effect. This shows that MAVS and IRF3 are essential for initiation of intracellular RNA-induced IFN responses in CHSE-214 cells. To elucidate viral interference with IFN induction pathways, the KOs were infected with Salmon alphavirus 3 (SAV3) and infectious pancreatic necrosis virus (IPNV). SAV3 infection in control and IRF7-1 KO cells yielded similar titers and no cytopathic effect, while IRF3 and MAVS KOs presented with severe cytopathic effect and increased titers 6 days after SAV 3 infection. In contrast, IPNV yields were reduced in IRF3 and MAVS KOs, suggesting a dependency on interactions between viral proteins and pattern recognition receptor signaling components during viral replication.

**Conclusion:**

Aside from more insight in this signaling in salmonids, our results indicate a possible method to increase viral titers in salmonid cells.

## 1 Introduction

The Atlantic salmon aquaculture industry in Norway has grown extensively over the last decades, but emerging and recurring diseases are still a problem. Many of these diseases are caused by viruses against which we lack effective vaccines (1). The interferon (IFN)– induced anti-viral state of host cells is a crucial component of successful protection against viral infection. For salmonid cells, antiviral responses in cell lines have shown a clear influence on viral replication (2–7). These cellular antiviral responses can be induced rapidly after activation of pathogen pattern recognition receptors (PRRs). Different PRRs recognize different pathogens or danger associated molecular patterns, and, in case of viral infection, these are often RNA molecules. Binding of their ligand leads to the activation of the PRRs, and the activation of signaling pathway(s) cumulates mainly in the production of IFNs (8). IFNs are cytokines that bind and activate extracellular IFN receptors on other cells or, as observed in rainbow trout, intracellular IFN receptors in the same cell (9). Finally, the IFN signaling pathway leads to the expression of IFN-stimulated genes (ISGs), most of which have anti-viral functions (10).

The PRRs consist of different families, such as Toll-like receptors (TLRs), nucleotide-binding oligomerization domain (NOD)-like receptors (NLRs), and Retinoic acid-inducible gene I (RIG)-I–like receptors (RLRs), whereas additional members have been described in recent years (11). The first and most extensively investigated PRRs are the TLRs. The number of TLRs identified in species varies quite a lot within vertebrates, with 13 TLRs described in mammals and 28 functional TLRs in teleosts (12, 13). TLRs are located on the cell membrane or in endosomal compartments and can recognize a wide range of molecules, such as LPS, flagellin, single- stranded RNA (ssRNA), double- stranded RNA (dsRNA), and CpG DNA (13). TLR signaling can occur through interrelated pathways, which usually include the adaptors Myeloid differentiation primary response 88 (MyD88) and/or TIR-domain-containing adapter-inducing interferon-β (TRIF), and leads to activation of one or more transcription factors, most notably, IFN regulatory factor (IRF) 3, IRF7, and NFκB, and, finally, IFN expression (13).

The RLR family consists of three cytosolic receptors: RIG-I, melanoma differentiation-associated protein 5 (MDA5), and Laboratory of Genetics and Physiology 2 (LGP2). These receptors recognize ssRNA or dsRNA. RIG-I and MDA5 ligand binding leads to activation of mitochondrial antiviral-signaling protein (MAVS) after interactions through caspase activation recruitment domains (CARDs) on the RLRs and MAVS (13), whereas LGP2 is suggested to have a function regulating the other RLRs. MAVS (also named CARDIF, IPS1, or VISA) contains a transmembrane domain that anchors it to the mitochondrial membrane, which is necessary for its function (14, 15). The signaling pathway downstream of MAVS activates similar transcriptions factors as the TLR pathway, followed by IFN transcription and induction of an anti-viral state through ISG expression (10).

Because these innate anti-viral responses are crucial for protection, it is expected that viruses have evolved ways to evade these responses (16). The naked dsRNA infectious pancreatic necrosis virus (IPNV) is a salmonid virus that potently inhibits the IFN response. IPNV infection *in vitro* does not induce IFN expression in certain cell types (10, 17), and several IPNV proteins have been shown to inhibit *ifna1* expression (18). Salmonid alphavirus (SAV), an enveloped ssRNA virus, is another highly pathogenic salmonid virus, but it strongly induces IFN responses in infected cell lines, in contrast to IPNV (19–22). An investigation into the role of key components of the PRR signaling that leads to IFN expression can help to gain more insight in these host–pathogen interactions. A knockout (KO) of these key components in cell lines through gene editing can shed light on their roles.

In recent years, gene editing has been hugely facilitated through revolutionary advances surrounding CRISPR-Cas. Originally discovered as an innate immune system in bacteria, CRISPR-Cas was soon developed into a cost-effective and fast way to introduce specific and targeted gene edits (23, 24). Although most protocols and reagents have been developed for use in mammalian systems, CRISPR-Cas gene edits have been performed in salmonids after injection in embryos (25), transfection of plasmids or ribonuclear proteins (RNPs) in cell lines (26, 27), or lentiviral delivery (28).

We investigated the differences between SAV and IPNV infections in CHSE-214 cells on an IFN activation level. More specifically, we evaluated the effect of PRR signaling upon stimulation or viral infection on ISG expression and viral growth in CHSE-214 cells by knocking out the RLR signaling molecule MAVS and the transcription factors IRF3 and IRF7-1. To this end, we developed a protocol for efficient CRISPR-Cas editing in CHSE-214 cells using RNP nucleofection and generated four KO cell lines: MAVS, IRF3, IRF7-1, and a IRF7-1/3 double KO. Because the CHSE-214 cell line seems to have limited TLR activity (29), these KOs would mainly affect RLR signaling. We evaluated the effect of the KOs on PRR signaling and viral growth through titration of virus, expression analysis of IFNs and ISGs, and promoter reporter assays to investigate activation of IFN and ISG promoters. Our results demonstrated that MAVS and IRF3 are essential for induction of IFN type I production in CHSE-214 cells, whereas the IRF7-1 KO did not affect IFN induction. The inhibition of IFN type I responses resulted in increased SAV3 titers, whereas IPNV titers were reduced. Those KO cell lines that showed an increased SAV3 replication could be useful for virus production in the industry or for research.

## 2 Materials and methods

### 2.1 Culture of cells and pathogens

Chinook salmon embryo cells (CHSE-214) (kindly provided by Bjørn Krossøy, Vaxxinova Norway AS) were grown in growth medium [L15 (PanBiotech) with 1% L-glutamine and 8% fetal bovine serum (FBS; HyClone)] at 20°C and passaged weekly at 2.5 × 10^6^ cells per 75- cm^2^ flask. CHSE-214 cells were single- cell– cloned through limited dilution by plating four cells per well in 96-well plates. Single colonies were transferred and expanded. Single- cell clone (Sc) 11 was used for transfections and included as negative control (NC) in later experiments.

IPNV (supplied by Vaxxinova Norway AS) was propagated on CHSE-214 cell culture at 18°. The cells were grown to about 80% confluence prior to infection, and IPNV was harvested at extensive cytopathic effect (CPE) after 2 days. The infected cell layer was freeze-thawed once before centrifugation at 5,000xg for 10 min to remove debris. The remaining supernatant was titrated by end-point titration, calculated by the 50% tissue culture infective dose (TCID_50_) method (30), and frozen in 1 ml of aliquots at −80°C until use in infection experiments.

SAV3 was provided by Øystein Evensen, Norwegian University of Life Sciences, and propagated on CHH-1 cell culture as described by 31. The supernatant was titrated on CHH-1 cells and frozen in 1 ml of aliquots at −80°C until use in infection experiments.

### 2.2 CRISPR-Cas editing

#### 2.2.1 Bioinformatics

We used genomic data from Atlantic salmon (*Salmo salar*) and rainbow trout (*Oncorhynchus mykiss*) to design sequencing primers on highly conserved regions between both species to sequence parts of the *mavs, irf3*, and *irf7-1* genes in CHSE-214 cells. These sequences were blasted [National Center for Biotechnology Information (NCBI)] against the Chinook salmon (*Oncorhynchus tshawytscha*) genome to verify the genes and obtain the gene IDs: *mavs* (112236223), *irf3* (112235560), and *irf7-1* (112252506).

We designed sgRNAs using either the Benching Guide RNA design tool or the Geneious CRISPR gRNA Design Software. sgRNAs were designed in batches of three with high predicted efficiency, low off-target effects, and homology with Atlantic salmon as criteria. Synthego produced the modified sgRNA (2′-O-Methyl at first three and last three bases and phosphorothioate bonds between the first three and last two bases). We investigated possible duplicate genes in chinook salmon by blasting (megaBLAST) the coding sequences of the targeted genes against the NCBI nucleotide collection for chinook salmon (assembly: Otsh_v2.0). Alignment and generation of phylogenetic trees was performed using Clustal Omega. The NCBI conserved domain search and TransMembrane prediction using Hidden Markov Models (TMHMM) tools predicted conserved and transmembrane domains respectively.

#### 2.2.2 Nucleofection of RNPs

The CHSE-214 wild-type (Wt) Sc 11 (NC) was nucleofected with CRISPR RNPs for genome editing using the 4D Nucleofector (Lonza). NC cells were passaged 1 day before nucleofection and seeded at 4 × 10^6^ cells per 75 -cm^2^ flask. RNP solution was prepared by mixing 1 μg of sgRNA and 2 μg of recombinant Cas9 (EnGen^®^ Spy Cas9 NLS, New England Biolabs) with nucleofector solution SE (Lonza) to a final volume of 10 μl, followed by 10 min of incubation at room temperature for complexing. NC cells (4 × 10^5^) were trypsinized, centrifuged at 300xg for 10 min, resuspended in 10 μl of nucleofector solution SE, mixed with the RNP solution, and added to a well in a 16-well Nucleocuvette strip. After nucleofection with program DS-137, the sample was incubated with 80 μl of OptiMEM (Gibco) for 10 min at room temperature and seeded in 12-well plates in growth medium. Transfection controls with pmaxGFP (Lonza) were evaluated after 2 days of incubation at 20°C.

#### 2.2.3 Editing efficiency and KO determination

Samples from transfected cells were lysed in a QuickExtract DNA extraction solution (LGC Biosearch) according to the manufacturer’s instructions, and purified PCR products of the target region were sequenced (Microsynth Seqlab). Sequencing chromatograms with superimposed peaks were analyzed using the online tool Tracking of Indels by Decomposition (TIDE) (32) for editing efficiency and indels present. Transfected pools with highest editing efficiency per target gene were used for single -cell cloning, and the Scs were evaluated by sequencing as described above. Scs with frameshift mutations in both targeted alleles were sampled and re-sequenced twice to verify the mutations. We used Geneious prime to check whether the mutations would result in premature stop codons in the open reading frame (ORF) and evaluated whether this disruption would lead to a KO of the targeted gene.

### 2.3 Poly I:C transfection and qPCR

NC and KO cells were seeded in 24- well plates with 2.5 × 10^5^ cells per well in 1 ml of growth medium with crosswise movement to spread the cells equally in the wells. One day later, cells were transfected with high– molecular weight (HMW) poly I:C by adding 100 µl of minimum essential media (MEM; Gibco), 1.2 μl of poly I:C (stock at 1 mg/ml, InvivoGen), and 3 μl of TransIT (Mirus) per well. RNA was isolated using the RNeasy kit (Qiagen) according to the manufacturer’s instructions 1 and 2 days after poly I:C transfection. Subsequently, cDNA was synthesized using the QuantiTect RT kit (Qiagen) according to the manufacturer’s instructions with 500 ng of RNA per 20 µl of reaction. cDNA was diluted 1:5 for use in qPCR reactions containing 6 µl of cDNA, 7.5 µl of 2× Fast SYBR^®^ Green Master Mix (Applied Biosystems), and 0.8 µl of each primer (5 μM stock). Taqman PCR reactions consisted of 5 µl of cDNA, 7.5 µl of 2x TaqMan universal master mix (Applied Biosystems), 0.18 µl of each primer (100 µM), 0.05 µl of probe (100 µM, 6FAM-BHQ1), and 2.09 µl of water. **Table 1** lists all primers used. The qPCR reactions were performed in 384-well plates under the following conditions: 95°C for 5 min and 45 cycles of 95°C for 5 s, 60°C for 15 s, and 72°C for 15 s (QuantStudio 6, Applied Biosystems). A melt curve stage was included to confirm the absence of nonspecific products in SYBR Green PCR reactions, primers and their references are presented in **Table 1**, and the efficiencies of tested primer pairs were between 90% and 110%. Relative expression was calculated using the delta Ct method with *elf2a* as a reference gene (37).

**Table 1:** List of primers used in this study with references to original publications of the primers.

Target	FW/RV	Sequence	Published in: *
*mavs* (1)	sgRNA	TGTCAGAAGGTGTAAGGCAA	
*mavs* (2)	sgRNA	CTGATGCTCCAACAGCTCCA	
*mavs* (3)	sgRNA	TTCCTTCTACCAGCTCTGAG	
*irf3*	sgRNA	TTCTAGGAAGGATTGCTCCG	
*irf7-1*	sgRNA	GCGAACAGATAAATAGTGGC	
*mavs*	FW	ACTGGACACCTAGGATCTCTGT	
	RV	CAGCAACAGGAGAAGGTGCT	
*irf3*	FW	ACTGGCTGATAGAACAAGTG	
	RV	ATGGGGGTCGTTTGAGTCCTTG	
*irf7-1*	FW	TCCCAGTTTACACAGGCTGTCA	
	RV	GGTGCTTTACCTCCTGTGGGT	
qPCR Target	FW/RV	Sequence	Published in: *
*elf2a*	FW	TGCCCCTCCAGGATGTCTAC	(33)
	RV	CACGGCCCACAGGTACTG	
*ifna*	FW	AAAACTGTTTGATGGGAATATGAAA	(29)
	RV	CGTTTCAGTCTCCTCTCAGGTT	
*ifnc*	FW	ATGTATGATGGGCAGTGTGG	(34)
	RV	CCAGGCGCAGTAACTGAAAT	
*allmx*	FW	TGCAACCACAGAGGCTTTGAA	(35)
	RV	GGCTTGGTCAGGATGCCTAAT	
*ifit5*	FW	GCTGGGAAGAAGCTTAAGCAGAT	(21)
	RV	TCAGAGGCCTCGCCAACT	
SAV3 *nsP1*	FW	CCGGCCCTGAACCAGTT	(36)
	RV	GTAGCCAAGTGGGAGAAAGCT	
*elf2a*	FW	TGCCCCTCCAGGATGTCTAC	(33)
	RV	CACGGCCCACAGGTACTG	
	Probe	AAATAGGCGGTATTGG	
*ifna (ifna1-2)*	FW	TGACTGGATCCGACACCACT	
	RV	ATCTCCTCCCATCTGGTCCA	
	Probe	AGCGCAGAATACCTTTCCCT	
*ifnc (ifnc1-4)*	FW	ATACCGCCAGATTGAAGAGAG	
	RV	CAGTCCTTCTGTCCTGATGAGATA	
	Probe	GGGCAGTGTGGATACCAGTG	
*mavs*	FW	GCTGATGAACTGAGGGCAGA	
	RV	GGTAGCAGCAGGTGAAGGAG	
	Probe	AGCACAACCAGAACAATCCCT	
*irf3*	FW	CAGGATTCCTGCAGCGATGA	
	RV	GTCGCCTTGAACCCTACCAT	
	Probe	ATTTTCAAGGCGTGGGCTGA	
*irf7*	FW	CTCCGAGGACGACCGTAAAA	
	RV	CCTTGTCAGTGGGATGCTCA	
	Probe	TATTCAGGGCATGGGCAGTG	

*If no reference is given, the primers were designed specifically for this investigation.

### 2.4 Luciferase assay

NC and KO cells were seeded in 96- well plates with 1.6 × 10^4^ cells per well in 100 µl of growth medium with 8% FBS and incubated for 1 day. Then, the cells were transiently transfected by replacing medium with neat L15 and adding 10 µl of transfection mix containing 100 ng of promoter reporter (firefly luciferase) construct, 10 ng of *Renilla* luciferase vector (Promega- Madison WI), and 0.3 µl of TransIT in MEM per well. Atlantic salmon *mx2*, *ifit5*, and *ifna1* promoter constructs (38) were investigated, whereas pGL3-basic was included as empty vector control. The promoters for *mx2* (35) and interferon induced protein with tetratricopeptide repeats 5 (*ifit5*) (21) were synthesized as GeneArt String fragments by ThermoFisher and cloned into HindIII-linearized pGL3 Basic using the Infusion HD cloning kit (Takara). One day after transfection, medium was replaced with 100 µl of neat L15 again, and transfection medium (10 µl of MEM with 0.3 µl of TransIT and 200 ng of stimulant) for HMW poly I:C or low –molecular weight (LMW) poly I:C was added. The medium was replaced with growth medium (8% FBS) 5 h post- transfection. All samples for the luciferase assays were set up in quadruplicates, and the constitutively expressing *Renilla* luciferase construct provided an internal control value to which the expression of the experimental firefly luciferase was normalized. Two days after transfection with stimulants, luciferase production was measured using the Dual-Luciferase Reporter Assay System (Promega, Madsion, WI) according to the manufacturer’s instructions. The results are presented as fold change in relative light units (RLU) by dividing the RLU of the stimulated samples by the average RLU of the corresponding non-stimulated samples.

### 2.5 Infections and CPE

#### 2.5.1 SAV3

NC and KO cells were seeded in 24 -well plates with 2.0 × 10^5^ cells per well in 1 ml of growth medium 1 day before infection. For each cell line, the number of cells per well was counted to calculate the amount of virus to be added to achieve the planned multiplicity of infection (MOI), and growth medium was replaced with 1 ml of infection medium with an MOI of 1 before incubation at 15°C. Supernatant for viral RNA qPCR and titration was sampled at 2 and 6 days post- infection (dpi). At 6 dpi, pictures were taken from selected wells to compare CPE. Cells were lysed for RNA extraction and expression analysis as described in 2.3 at 2 and 6 dpi.

#### 2.5.2 IPNV

KO cells were seeded in 24- well plates with 1.25 × 10^5^ cells per well in 1 ml of infection medium (L15 with 1% L-glutamine and 2% FBS) 1 day before infection. For each cell line, the number of cells per well was counted to calculate the amount of virus to be added to achieve an MOI of 0.01, and IPNV was added to the wells before incubation at 18°C. At 2 dpi, supernatant for titration was sampled, pictures were taken from selected wells, and the cell layer was sampled. Cell layers were either fixed with 4% formaldehyde for crystal violet staining or lysed for RNA extraction and qPCR as described in 2.3. Expression analysis was performed at 1 and 2 days after infection on infected cells that were originally seeded at 2.5 × 10^5^ cells per well. Formaldehyde-fixed cells were washed with phosphate- buffered saline (PBS) and stained with 1% crystal violet in PBS to quantify CPE. After 10 min of incubation at room temperature, the wells were carefully washed three times with H_2_O and dried. Non-specific staining was removed from well walls, and the remaining crystal violet eluted by shaking for 5 min with 200 µl of elution buffer (50% ethanol with 0.05 M sodium citrate and 0.05 M citric acid). The OD_590_ was determined using a Sunrise absorbance reader (Tecan).

### 2.6 Titrations

IPNV supernatant samples were titrated by end-point titration on CHSE-214 cells with eight wells per dilution, and CPE was scored after 14 days. SAV3 supernatant samples were titrated on MAVS KO CHSE-214 cells (described in Section 3.1) with eight wells per dilution. These cells showed clear CPE after SAV3 infection, and CPE was used to score the titration 14 dpi after we determined that this scoring method was as reliable as staining with anti-SAV antibodies according to Strandskog et al. (39) (unpublished results). TCID_50_/ml was calculated following the method of Reed and Muench (30).

### 2.7 Viral RNA: cDNA and qPCR

The viral RNA from SAV3- infected NC and KO cell supernatants was isolated using the QIAamp Viral RNA mini kit (Qiagen) according to the manufacturer’s instructions, with the exception that no carrier RNA was used. Subsequently, the QuantiTect RT kit (Qiagen) was used for cDNA synthesis according to the manufacturer’s instructions with 12 µl of isolated RNA per reaction. qPCR reactions contained 6 µl of cDNA (1:5 diluted), 7.5 µl of 2× Fast SYBR^®^ Green Master Mix (Applied Biosystems), and 0.8 µl of each primer (5 μM stock). The following conditions were used for the amplification: 95°C for 5 min and 45 cycles of 95°C for 5 s, 60°C for 15 s, and 72°C for 15 s (7500 Fast Real-Time PCR System, Applied Biosystems). Melting curves were used to confirm the absence of nonspecific products, and the used primers have previously been tested (see **Table 1** for references). A dilution series of an amplicon with known concentration was included to generate a standard curve for calculation of DNA copies per sample.

### 2.8 Statistics

We performed statistical tests in GraphPad Prism version 8.4.1. Outliers were removed using the ROUT test with Q = 1%. Welch’s ANOVA with Dunnett’s T3 multiple comparisons test (α = 0.05) was performed to find significant differences between the NC and the KOs. The data for IPNV titrations were not normally distributed, and an ANOVA (Kruskal–Wallis) with Dunn’s multiple comparisons test was used instead. One-sample *t*-tests against the theoretical mean of 1 (no change) were used to evaluate gene induction in poly I:C– stimulated NC.

## 3 Results

### 3.1 Efficient CRISPR-Cas editing in CHSE-214 cells through RNP nucleofection

Because our gene editing protocol involved isolating and infecting Scs from an edited pool, we investigated whether Wt Scs from the CHSE-214 cell line yielded different IPNV titers after infection. **Supplementary Figure 1** shows that there is a significant difference in IPNV titers between individual clones and the CHSE-214 pool. To eliminate the risk of observing differences between gene-edited cell lines resulting from variation between the original cells in which the gene-edits are introduced, Sc 11 (NC), which had a significantly lower IPNV replication but propagated well, was used for all subsequent gene editing experiments and included as a NC in later experiments.

Gene editing efficiencies were quite variable between the sgRNAs tested. The final sgRNAs resulted in editing efficiencies of 76%, 23%, and 73% for *mavs*, *irf3*, and *irf7-1*, respectively, based on decomposition of sequencing chromatograms by the TIDE webtool. We isolated Scs from these edited NC pools and picked one clone per gene edit for further analysis. The indels in all selected Scs for IRF3 and IRF7-1 KOs led to premature stop codons within the first 50 amino acids, which is within the DNA binding domain, visualized in **Supplementary Figure 2**. The mutations in the alleles of the MAVS KO led to premature stop codons after 124 and 125 amino acids, respectively (**Supplementary Table 1**). The CARD domain would be mostly intact in these truncated proteins, but the C terminal transmembrane domain that is also essential for MAVS function in both human (14) and Atlantic salmon (15) is missing. In conclusion, the verified mutations in the presented Scs lead to KO of the genes of interest.

A megaBLAST search of the targeted coding sequence revealed no duplicate genes for *irf3* and *irf7-1* in the chinook salmon genome (assembly Otsh_v2.0) but identified a possible duplicate *mavs* gene. The work of another group has identified a duplicate gene of *irf7-1*, which is not yet annotated (personal communication, Dr. B. Collet). The *mavs* duplicate gene (Gene ID: 112237596) has a 67% homology on the RNA level with our targeted gene (47% on protein level) and is not targeted by the used sgRNAs. The putative duplicate *mavs* gene contains a conserved death domain, which could indicate a CARD domain, and an N-terminal transmembrane domain and could therefore have a MAVS-like function (see **Supplementary Material** for more details).

Off-target analysis with CCTop did not yield any possible off-target effects in other genes with less than three mismatches. Furthermore, a BLAST search of the sgRNA sequences on NCBI only returned high identity results in other species or in the targeted genes in chinook salmon. Thus, the chance of off-target effects seemed to be quite low, also considering the temporary activity of the Cas9 protein due to delivery in RNP format.

### 3.2 MAVS and IRF3 KOs inhibit induction of ISG expression

**Figure 1:**
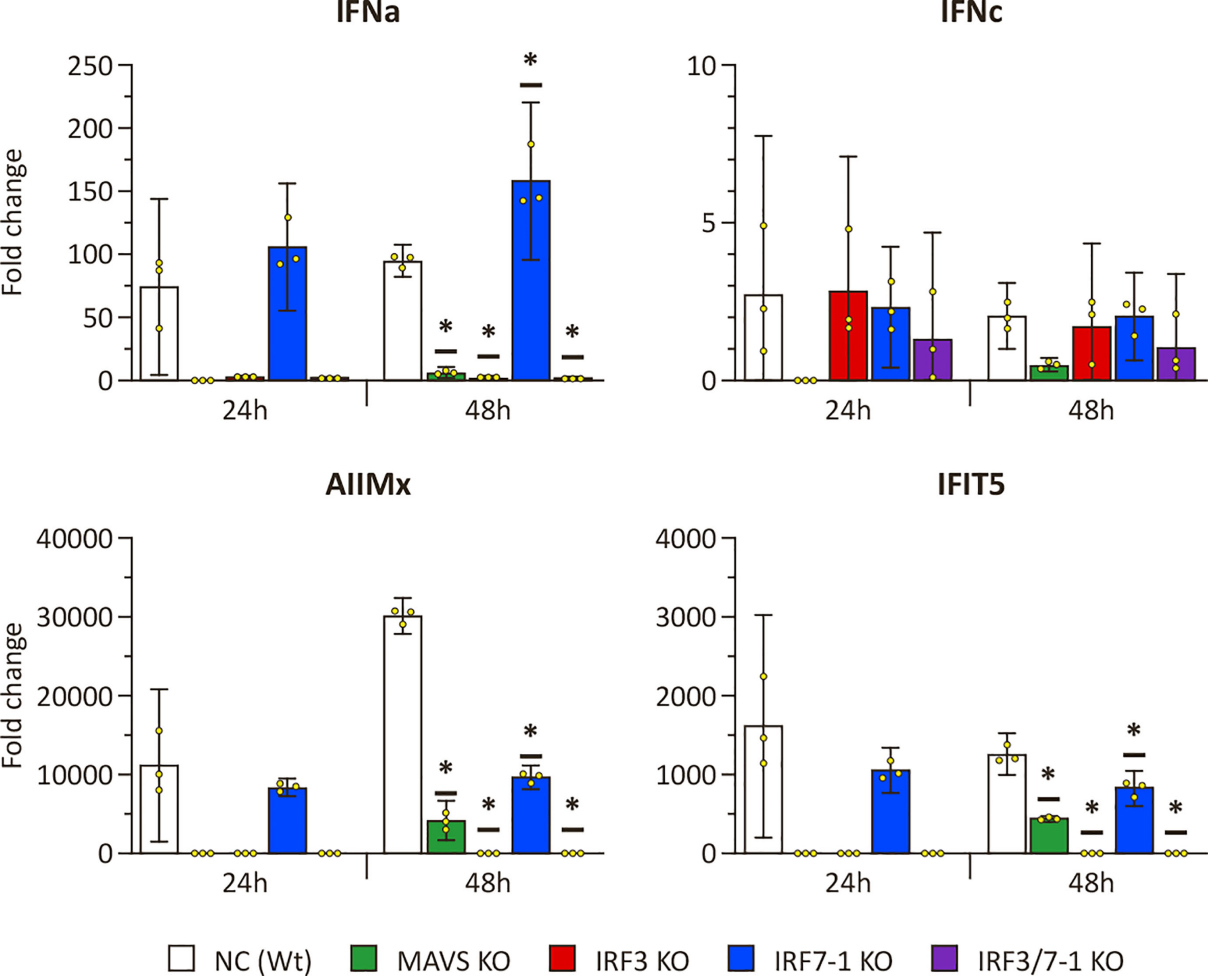
Expression of IFNs and ISGs in MAVS, IRF3, and IRF7-1 KO CHSE-214 cells 24 and 48 h after HMW poly I:C transfection measured by quantitative PCR. The graphs show the fold change of expression compared to non– poly I:C– transfected controls and normalized against *elf2a*. Values of the triplicates visualized as dots, and error bars indicate 95% confidence interval. (*) Statistically significantly different from the wild-type NC.

To investigate the effect of the KOs on PRR signaling, we evaluated the ability of KO cells to express IFNs and ISGs upon intracellular poly I:C stimulation after 24 and 48 h (**Figure 1**). The expression of *mx* and *ifit5* genes, both ISGs with antiviral activity, was successfully induced at both time points after poly I:C transfection in the Wt NC (**Figure 1**). The IRF7-1 KO showed a similar induction as the Wt cells at 24 h, which was reduced at 48 h for the ISGs, whereas the *ifna* expression was increased. In stark contrast, for both the IRF3 and IRF7-1/3 KOs, this induction was completely abolished at both time points. In the MAVS KO, the induction was reduced, although not as extreme as the IRF3 and IRF7-1/3 KOs at 48 h. A similar trend was observed for *ifna*, where the expression was induced in NC and the IRF7-1 KO, (but) abolished in the IRF3 and IRF7-1/3 KOs, whereas the MAVS KO showed reduced *ifna* transcript levels. In contrast to *mx* and *ifit5*, *ifna* induction in IRF7-1 KO at 48 h was higher than that in the NC. We did not observe a significantinduction of *ifnc* at these time points, although the MAVS, IRF3, and IRF7-1/3 KOs had a slightly lower induction at 48 h (**Figure 1**). These results indicated a disruption of PRR signaling in MAVS and IRF3 KO cells.

### 3.3 Reduced ISG promoter activation in MAVS and IRF3 KO cells after PRR stimulation

**Figure 2:**
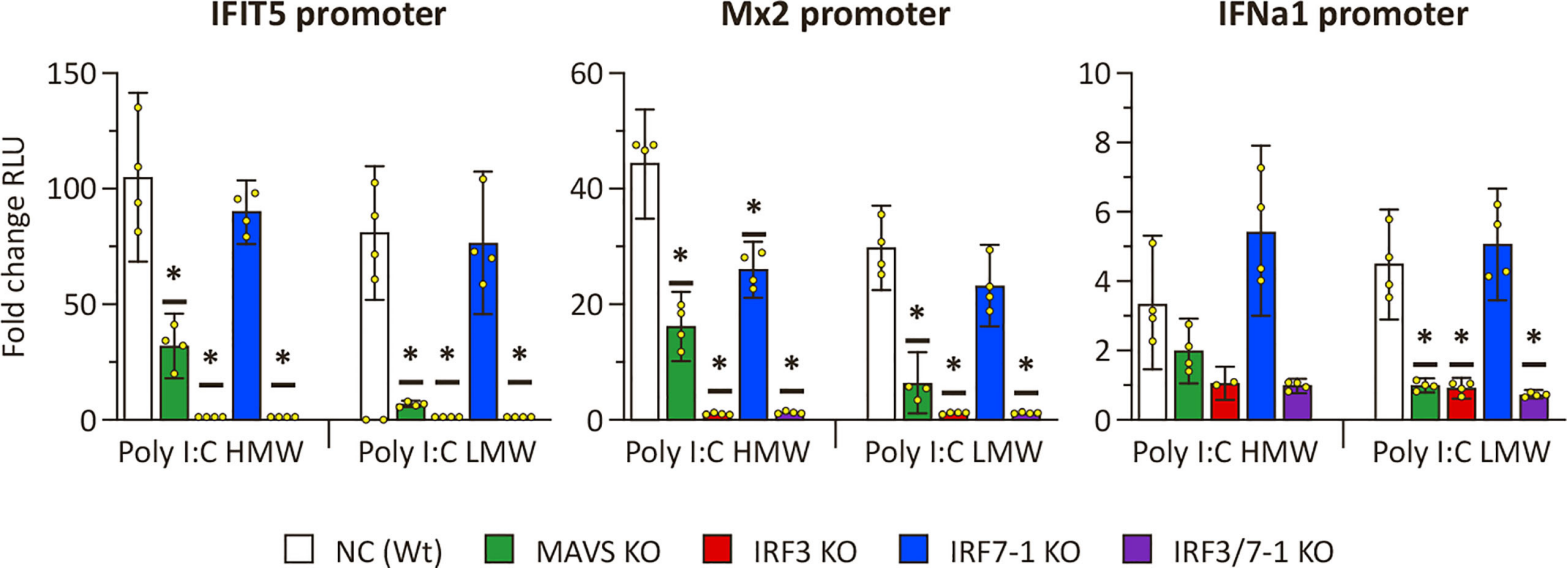
*ifit5*, *mx2*, and *ifna1* promoter activation in MAVS, IRF3, and IRF7-1 KO CHSE-214 cells 48 h after HMW or LWM poly I:C transfection. The graphs show the fold change of RLU (normalized against co-transfected Renilla plasmid) compared to non– poly I:C– transfected controls. Values of the quadruplicates visualized as dots, and error bars indicate 95% confidence interval. (*) Statistically significantly different from the wild-type NC. These data represent one of three repeated experiments which gave reproducible results.

To confirm the disruption of PRR signaling by MAVS and IRF3 KOs as evidenced by the expression results, we investigated ISG promoter activation upon intracellular poly I:C stimulation of the KO cells (**Figure 2**). All investigated promoters (*ifit5*, *mx2*, and *ifna*1) showed a clear activation in NC and the IRF7-1 KO 48 h after both HMW and LMW poly I:C transfection compared to non-stimulated controls (**Figure 2**). In the IRF3 and IRF7-1/3 KOs, this activation was almost completely absent, whereas the MAVS KO resulted in a reduced activation. Both the empty vector (pGL3-basic) and non-stimulated controls showed very low background activation (**Supplementary Figure 3A**), and a second experiment confirmed the inhibition of activation in the MAVS, IRF3, and IRF7-1/3 KOs (**Supplementary Figure 3B**). Together, these data confirm that the MAVS and IRF3 KOs inhibit IFN responses after intracellular poly I:C stimulation of the cells, whereas the IRF7-1 KO does not have a strong inhibitory effect on the IFN response.

### 3.4 Increased virus titers and CPE after SAV3 infection of MAVS and IRF3 KO cells

**Figure 3:**
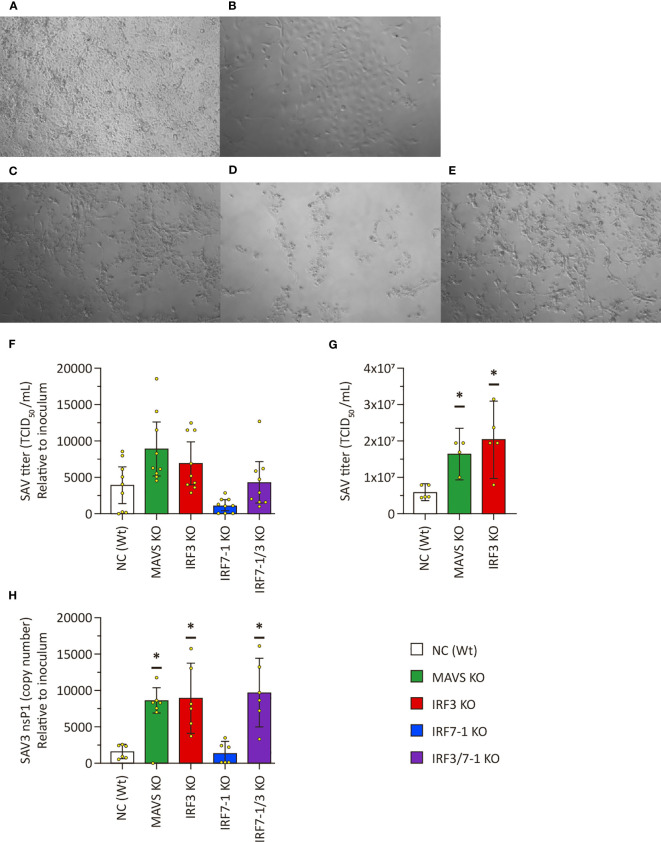
Salmonid alphavirus 3 (SAV3) growth on KO CHSE-214 cells. **(A–E)** Representative pictures of CPE on SAV3 -infected cells 6 dpi: NC **(A)**, IRF7-1 KO **(B)**, IRF3 KO **(C)**, IRF7-1/3 KO **(D)**, and MAVS KO **(E)**. **(F)** SAV3 titers in supernatants of infected KO cells relative to the used inoculum at 6 dpi. **(G)** SAV3 titers in supernatants of infected KO cells with the same inoculum at 6 dpi. **(H)** SAV3 *nsp1* transcript levels in supernatant of infected KO cells relative to the used inoculum at 6 dpi. Presented as # DNA copies (SAV3 *nsp1*) in 6 µl of cDNA from 12 µl of RNA isolate divided by the viral titer of the inoculum. Values of triplicates from three (viral titer F), one (viral titer G), or two (viral RNA H) experiments visualized as dots, and error bars indicate 95% confidence interval. (*) Statistically significantly different from the wild-type NC.

After having investigated the impact on the different KOs on antiviral response assays, we aimed to understand their effects on virus replication. We therefore tested viral growth for the enveloped ssRNA virus SAV3 and the naked dsRNA IPNV to assess the impact of the different KOs on their growth. Both viruses not only are sensitive to the antiviral effects of type I IFNs but also possess strategies to counteract/modulate IFN activity (16)). We first infected the MAVS, IRF3, and IRF7-1 KOs with SAV3 to investigate whether and how the disruption in the PRR signaling pathway would affect virus growth. Some CPE was present in NC at 6 dpi (**Figure 3A**), which was in line with previous observations that SAV3 infection usually results in minor CPE in CHSE-214 cells. The IRF7-1 KO cell layer similarly exhibited some CPE (**Figure 3B**). In contrast, SAV3 infection resulted in massive CPE in the MAVS, IRF3, and IRF7-1/3 KOs, which suggests an increased SAV3 replication (**Figures 3C–E**). The appearance of clear CPE on MAVS and IRF3 KOs allowed for titration on these cells without staining with antibodies as described by Strandskog et al. (39). Visual CPE scoring of titrations on MAVS KO cells compared very well with scoring based on antibody staining (data not shown). As a result, we used the MAVS KO cells to determine virus titers.

SAV3 titers in supernatant of the infected IRF7-1 KO cells were slightly (non-significantly) reduced compared to NC (**Figure 3F**), but this could be due to the apparent difference in confluence leading to less cells being available for production of viral particles. From three separate experiments, we found a clear increase in viral titers in the MAVS and IRF3 KOs in (**Figure 3F**). An additional experiment with just these KOs further confirmed this (**Figure 3G**). Viral RNA in the supernatant as determined by qPCR mimics the trend seen for the titration results: an increase in the MAVS and IRF3 KOs, a slight reduction in the IRF7-1 KO, and IRF7-1/3 between IRF3 and IRF7-1 results (**Figure 3H**).

**Figure 4:**
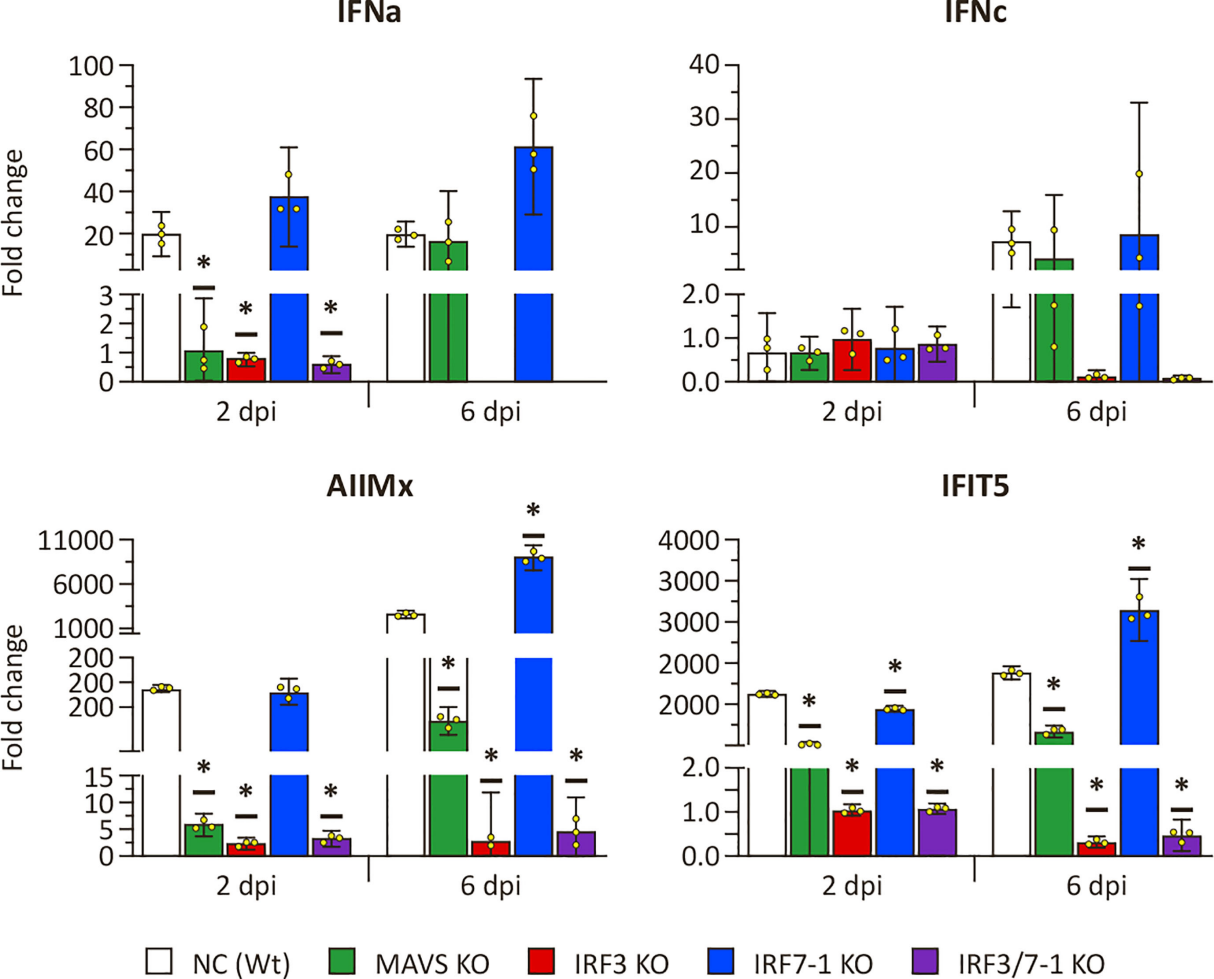
Expression of type I IFN and selected ISGs in MAVS, IRF3, and IRF7-1 KO CHSE-214 cells determined by quantitative PCR, 2 or 6 dpi after Salmonid alphavirus 3 (SAV3) infection. The graphs show the fold change of expression compared to non-infected controls and normalized against *elf2a*. Values of the triplicates visualized as dots, and error bars indicate 95% confidence interval. (*) Statistically significantly different from the wild-type NC.

Because the MAVS and IRF3 KOs had a clear effect on the expression of ISGs after intracellular poly I:C stimulation, we investigated whether a similar effect could be observed after SAV3 infection. To this end, we measured the expression of the same genes at 2 and 6 dpi (**Figure 4**). *Mx*, *ifit5*, and *ifna* genes were induced in NC and the IRF7-1 KO (**Figure 4**). In contrast, IRF3 and IRF7-1/3 KOs showed no elevated levels of these genes after infection, and, for the MAVS KO, induction was reduced compared to Wt (**Figure 4**). These observations were comparable to the poly I:C stimulation results (**Figure 1**). The later time point (6 dpi) showed a general slight increase in induction of *mx*, *ifit5*, and both measured IFNs in NC, the MAVS KO, and the IRF7-1 KO compared to 2 dpi. *ifnc* was not induced at the early time point, as was seen for poly I:C, but showed upregulation (albeit not significant) in NC, the MAVS KO, and the IRF7-1 KO at the later time point (6 dpi). This was in contrast to the poly I:C stimulated cells where we did not observe a clear induction (**Figure 1**). This increase of *ifnc* after SAV infection was absent in the IRF3 and IRF7-1/3 KOs, similar to the other investigated genes.

### 3.5 Reduced IPNV titers in MAVS and IRF3 KO cells

**Figure 5:**
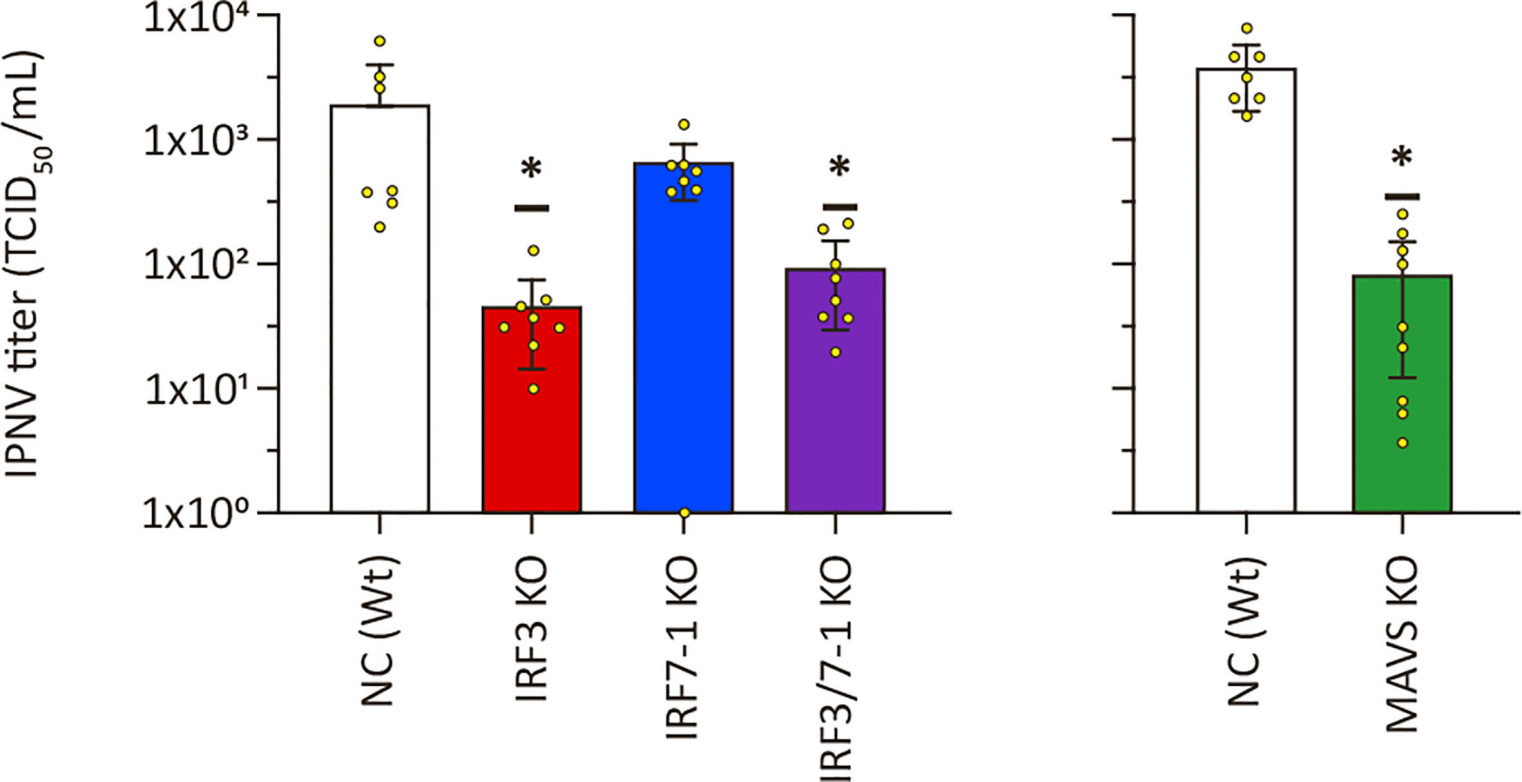
Infectious pancreatic necrosis virus (IPNV) replication in MAVS, IRF3, and IRF7-1 KO CHSE-214 cells. IPNV titers in supernatants of infected KO cells 2 dpi. Values of triplicates from three experiments visualized as dots, and error bars indicate 95% confidence interval. (*) Statistically significantly different from the wild-type NC.

To investigate whether the increased viral titers was a common feature on viral replication for MAVS and IRF3 KO cells, we infected the cells with another virus, IPNV. We evaluated IPNV replication on the KO clones by titrating the supernatant harvested at 2 dpi from infected KO cells. Inactivation of IRF7-1 did not lead to a significant difference, although viral titers were slightly lower (**Figure 5**). Interestingly and in contrast to the increase in titers seen for SAV3, IPNV titers were significantly reduced in MAVS, IRF3, and IRF7-1/3 KO cells compared to Wt controls (**Figure 5**). Determination of CPE through crystal violet staining showed a significantly reduced CPE for the IRF3 KO, but not for the other clones (**Supplementary Figure 4**). No loss of cells due to CPE was detectable in the MAVS KO cells or the corresponding NC at the time of harvest, so these results were not presented. In summary, disrupting PRR signaling by inactivation of IRF3 or MAVS reduced IPNV replication.

**Figure 6:**
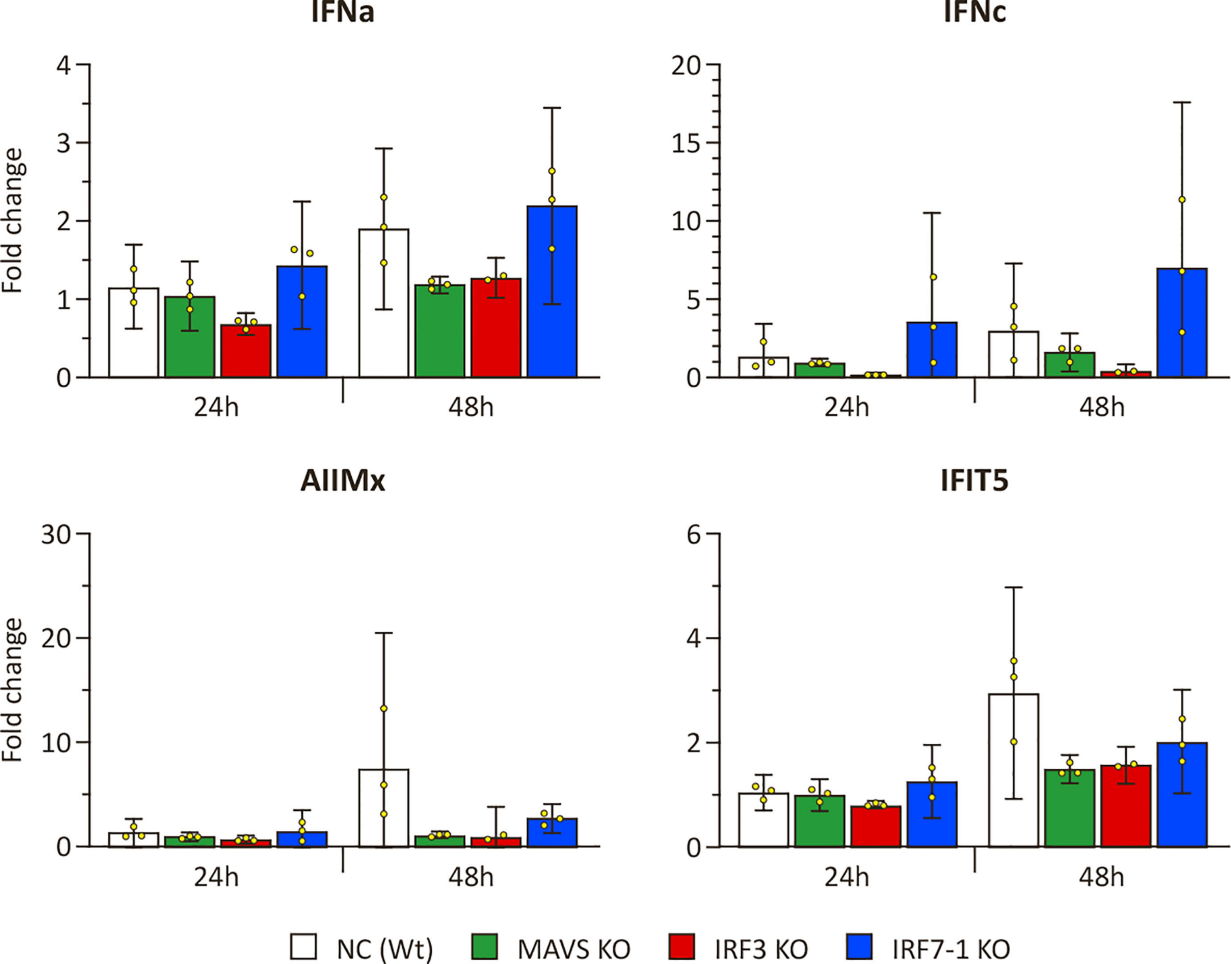
Expression in MAVS, IRF3, and IRF7-1 KO CHSE-214 cells 1 or 2 dpi after infectious pancreatic necrosis virus (IPNV) infection. The graphs show the fold change of expression compared to non-infected controls and normalized against *elf2a*. Values of the triplicates visualized as dots, and error bars indicate 95% confidence interval. These data represent one of the two repeated experiments that gave reproducible results.

We evaluated ISG and IFN transcript levels in IPNV -infected NC and KO cells to investigate the observed differences in effect on viral growth with SAV3. Induction of *ifit5* and *mx* was very low after IPNV infection (**Figure 6**) when compared to the induction after SAV3 infection (**Figure 4**) but seems to be present, nonetheless. In particular, after 48 h, we observed an induction in the Wt NC, whereas this was reduced in the MAVS and IRF3 KOs. The induction of *ifit5* and *mx* in IRF7-1 KO was closer to the NC, as generally observed in our other experiments. *ifna* was slightly, although not significantly, induced after 48 h, which was mainly noticeable due to the apparent reduced induction in the MAVS and IRF3 KOs (**Figure 6**). Interestingly, *ifnc* was slightly induced in the NC and IRF7-1 KO with a smaller induction in the MAVS KO, as seen after SAV3 infection.

## 4 Discussion

### 4.1 Efficient CRISPR-Cas editing in CHSE-214 cells using nucleofection of RNPs

The type I IFN response is the immune system’s early weapon against viral infections. It can be triggered in many cell types by detection of viral nucleic acids through the activation of different PRRs (13). RLRs detect dsRNA in the cytosol, and the adapter protein MAVS is essential for their signaling. IRF3 and IRF7 are master transcription factors for the type I IFN response in mammalian species and are also known to be important regulators of IFN responses in teleosts (40). In this study, we employed a functional genomics approach to elucidate the roles of salmon MAVS, IRF3, and IRF7-1 in the antiviral responses against viruses in the CHSE-214 cell line. We successfully generated KO clones for MAVS, IRF3, and IRF7-1, as well as a double KO for IRF7-1/3. This was possible due to the use of nucleofection to deliver RNPs for CRISPR-Cas editing that enabled us to obtain high editing efficiency. Our results were comparable with the editing efficiencies that (27) obtained in salmonid cells using another optimized RNP protocol (slightly over 70%).

Initial testing on IPNV infection of IRF7-1 edited Scs generated from the original CHSE-214 cell line showed significant differences in viral titer between several obtained IRF7-1 KO clones and between a Wt clone and the original CHSE-214 cell line (results not shown). This could have been a result of off-target edits or differences between the single parent cells. However, the chance of all tested clones having off-target edits influencing IPNV replication is probably not that high. We confirmed that different Wt Scs can lead to differences in IPNV replication and used one of these clones to develop KO clones from the same parental clone to reduce any possible effect from different parental cells. This heterogenicity of Wt cell lines has been confirmed in a mammalian setting, and the use of monoclonal cells for gene editing was found to lead to less variability (41).

### 4.2 MAVS and IRF3 KO inhibit PRR signaling

The MAVS, IRF3, and IRF7-1 KOs led to different effects on IFN and ISG induction after intracellular poly I:C stimulation: full inhibition in the IRF3 KO clones, reduced inhibition in the MAVS KO clone, and induction that is most comparable to the Wt in the IRF7-1 KO. The results from both the expression and promoter activation experiments indicate that IRF3, and not IRF7-1, is an essential transcription factor for IFN type I induction in CHSE-214 cells. The clear difference of KO effects between IRF3 and IRF7-1 is notable, especially because both these transcription factors contribute to PRR signaling and show synergetic activity in Atlantic salmon (40). Nonetheless, IRF3 was found to be a stronger activator of the *ifna1* promoter in Atlantic salmon TO cells than IRF7-1 (40). The identification of an additional copy of IRF7 in Chinook salmon, as earlier found in Atlantic salmon (personal communication, Dr. B. Collet), could explain the lack of phenotypical change in our KO. Although the role of the second IRF7 gene remains to be elucidated, our results suggest that the herein inactivated IRF7-1 has a less prominent role than the other duplicate. In mammals, high basal expression of IRF7 is largely restricted to immune cells, such as B cells and plasmacytoid dendritic cells (42). Because CHSE-214 cells are non-lymphocyte lineage cells, they would not express IRF7, and we would not expect a KO of IRF7 to have an effect on these cells. However, our results show comparable basal expression of *irf3* and *irf7* transcripts in the CHSE-cells (**Supplementary Figure 5**), which is not expected according to the mammalian paradigm. We did observe higher *ifr3* mRNA levels compared to *irf7* after stimulation (**Supplementary Figure 6**), which suggests that IRF3 has a more prominent role than IRF7. Our results fit in a model where IRF3 is essential in initiating IFN expression, whereas IRF7 enhances these responses and is more tightly regulated (43, 44).

A less pronounced difference in KO effect was found between MAVS and IRF3. The results of expression induction and promoter activation for multiple genes after intracellular poly I:C stimulation showed a complete inhibition of induction in the IRF3 KO clones, whereas the MAVS KO clone at times only led to a partial inhibition. This difference could be due to the PRR pathways that these genes have a function in. MAVS is a major component of the RLR pathway but has no major function in other PRR pathways (11). IRF3, in contrast, is involved in signaling of several PRRs (13, 45). Intracellular poly I:C stimulates not only RLRs but also certain NLRs and TLRs (13). The IRF3 KO would affect all these pathways, whereas the MAVS KO only affects the RLR pathway. A second explanation for the difference in effect between the MAVS and IRF3 KOs would be the presence of a duplicate *mavs* gene in salmonids. We identified a putative duplicate *mavs* gene with 67% homology on mRNA level (see **Supplementary Material**). Domain predictions and synteny in Atlantic salmon, rainbow trout, and chinook salmon indicate that this gene probably arose from *mavs* after a duplication event and could possess MAVS like function. The low level of homology on protein level (47%), however, generates doubts on how much of the original function is kept. If some of the original function is retained in the duplicate gene, then this could account for the observed incomplete inhibition of PRR signaling, because the used sgRNAs did not target the duplicate gene. Further investigation could elucidate whether the duplicate *mavs* gene has a function and how similar this function is to the original *mavs* gene.

Activation of the *ifna1* promoter led to much lower RLU values (**Supplementary Figure 3**) compared to the other used promoter constructs. These values are comparable to the values of an earlier publication using the same construct (38). This suggests that the *ifna1* promoter is activated at much lower levels than the ISG promoters from *mx2* and *ifit5* after poly I:C stimulation. IFNs are signaling cytokines whose signal is amplified in receiving cells and that need to be carefully regulated to avoid extreme immune responses (46). In contrast, Mx2 and IFIT5 have a direct anti-viral activity for which they need to be expressed at sufficient levels. It is thus logical that the strongly regulated IFN promoter is less activated as the *mx2* and *ifit5* promoters, which is also in line with our expression results.

The early induction of *ifna*, *ifit5*, and *mx* genes and the missing induction of *ifnc* after stimulation suggests that IFNa, and not IFNc, is responsible for initial ISG transcription in CHSE-214 cells in response to poly I:C stimulation. Because chinook salmon, like other salmonids, possesses multiple IFN genes and our primers amplify mRNA of several genes based on our bioinformatic analyses (unpublished results), additional IFN genes could be involved in the IFN responses initiated by poly I:C transfection. A complete IFN gene expression analysis would be an entire investigation on its own. Still, the late induction of *ifnc* by SAV3 at 6 dpi and the minor induction after IPNV infection indicates a differential expression pattern of these IFN genes.

### 4.3 PRR signaling disruption increases SAV3 replication in CHSE-214 cells while decreasing IPNV replication

Our results clearly show that disrupting PRR signaling positively affects SAV3 replication in CHSE-214 cells, as illustrated by the increased CPE, viral titers, and viral RNA. In addition, inactivation of MAVS and IRF3 abolished the activation of antiviral genes such as *ifn*, *mx*, and *ifit5* that we observed in SAV3- infected Wt cells. Still, the effect of MAVS and IRF3 KOs on viral replication seems to be dependent on the combination of virus and cell type. Our results show a different effect of MAVS and IRF3 KOs on the replication of two different viruses, SAV3 and IPNV, on the same cell line. The mammalian literature contains more examples of diverging effects of MAVS KO on viral replication where different viruses or different cell types/tissues have been investigated (47, 48).

One unexpected finding is that SAV3 replicated equally well in the MAVS and IRF3 KO clones, whereas the disruptive effect of the IRF3 KO on PRR signaling was more pronounced. It is possible that, after IFN responses have been reduced below a critical level, the viral replication is not affected by any further reduction. This would mean that SAV3 already replicates at peak efficiency after partial inhibition of IFN responses and that complete inhibition is not necessary for elevated SAV3 replication. Finally, a difference in replication kinetics between the KOs could result in an over- or underestimation of the titers during a comparison at one time point. We showed a difference in dynamics, but there might still be a comparable final titer if later time points would be analyzed.

The fact that inactivation of PRR signaling did not increase IPNV titers fits well with a model wherein IPNV can inhibit PRR signaling *in vitro*. This model is based on several observations. Although IFN responses were found to be induced by IPNV in tissues of infected Atlantic salmon (49), primary macrophages (17), and RTG-2 cells (50), it is usually not induced after IPNV infection in the cell lines CHSE-214, TO, and SHK-1 (10, 18, 19, 51). It is interesting that we observed a very minor induction of *ifna*, *mx*, and *ifit5* expression after *in vitro* IPNV infection, in contrast to these earlier findings. This induction was mainly visible due to comparison with the non-induced MAVS and IRF3 KO clones, which could be why it has not been registered earlier. In addition, it has been demonstrated that several IPNV proteins interact with and inhibit multiple components taking part in IFN and ISG induction with a profound effect on MAVS-mediated activation of the *ifna1* promoter (16, 18). Overall, the rapid and extensive CPE that IPNV causes on CHSE-214 cells suggests that antiviral responses do not strongly inhibit the viral infection in these cells or that the viral replication is fast enough to overwhelm the responses. Our findings that KO in PRR signaling does not increase IPNV replication in CHSE-214 cells further strengthen this model. The interaction of IPNV proteins with PRR signaling components could offer an explanation why IPNV replication decreased in the MAVS and IRF3 KOs. During the evolution of IPNV to combat the antiviral responses, the virus could have become partially dependent on these interactions, in addition to just inhibiting the antiviral responses. This dependency on interactions with host components would not be unexpected considering virus–host co-evolution and can explain the reduced viral replication after our KOs of host PRR signaling.

Our results clearly indicate that MAVS and IRF3 are interesting targets to improve SAV3 growth on the CHSE-214 cells. Using the Wt NC for gene editing, we managed to increase the production some two to three times. Although this is a modest increase, the optimal timing of harvest could be different for the differently edited clones. Finding the ideal time point of harvest could increase the obtained SAV3 titers, but higher titers should also be achievable by selecting other Sc from CHSE-214 for gene editing, possibly leading to a new efficient production substrate for SAV3. The clear CPE on MAVS and IRF3 KO CHSE-214 clones also made it possible to use visual scoring to read-out titrations on these cells. Previously, titrations of SAV3 on CHSE-214 cells would be read out after staining with anti-SAV antibodies (39), which takes more time and requires expensive antibodies. The use of MAVS or IRF3 KO clones for titration of SAV3 samples thus reduces costs for experiments that would otherwise titrate on the CHSE-214 cell line.

We have successfully shown that viral replication in CHSE-214 cells is affected by disrupting PRR signaling with CRISPR-Cas –induced MAVS or IRF3 KOs. KO of IRF7-1 showed no or minor effects on PRR signaling after internal poly I:C stimulation or viral infections. In contrast, KOs of IRF3 completely blocked the induction of type I IFNs and IFN-induced ISGs, demonstrating the vital importance of IRF3 for IFN induction in non-lymphoid salmonid cells. These responses were also reduced in MAVS KO clones, suggesting that RIG-I signaling is essential in CHSE-214 cells. However, because IFN-induction was not totally abolished in the MAVS KO, other PRR signaling pathways are likely involved in dsRNA- mediated signaling in these cells. The effect of PRR signaling disruption was pathogen dependent, with SAV3 replicating better in MAVS and IRF3 KO clones, but IPNV titers being reduced. Future research could focus on the effect of KO of MAVS and IRF3 on the replication of additional viruses, infectious salmon anemia virus, for example, and in other cell lines. This research could lead to enhanced substrates to produce salmonid viruses and thus lower costs for research and vaccine production. In addition, the edited cell lines might even support replication of viruses that cannot be cultivated on currently available cell lines. Finally, deeper insight in the PRR pathways affecting different viruses could be used to generate leads for new adjuvants in the form of PRR ligands for viral vaccines.

## Data availability statement

The raw data supporting the conclusions of this article will be made available by the authors, without undue reservation.

## Author contributions

YW, JK, and JJ conceived and designed research. YW, AA, HN, and LG-T performed research and analyzed data. AA helped design experiments. YW and JJ wrote the paper. All authors reviewed and approved the manuscript.

## References

[1] Adams A. Progress, challenges and opportunities in fish vaccine development. Fish shellfish Immunol (2019) 90:210–4. doi: 10.1016/j.fsi.2019.04.066 31039441

[2] Robertsen Børre Bergan V Røkenes T Larsen R Albuquerque A. Atlantic Salmon interferon genes: cloning, sequence analysis, expression, and biological activity. J Interferon Cytokine Res (2003) 23(10):601–12. doi: 10.1089/107999003322485107 14585200

[3] Ooi EiL Verjan N Hirono I Nochi T Kondo H Aoki T. Biological characterisation of a recombinant Atlantic salmon type I interferon synthesized in escherichia coli. Fish shellfish Immunol (2008) 24(5):506–13. doi: 10.1016/j.fsi.2007.10.004 18329900

[4] Berg K Svingerud T Sun B Robertsen Børre. An antiserum against Atlantic salmon *IFNa*1 detects IFN and neutralizes antiviral activity produced by poly I:C stimulated cells. Dev Comp Immunol (2009) 33(4):638–45. doi: 10.1016/j.dci.2008.11.005 19063917

[5] Xu C Guo T-C Mutoloki S Haugland Øyvind Marjara IS Evensen Øystein. Alpha interferon and not gamma interferon inhibits salmonid alphavirus subtype 3 replication *in vitro* . J Virol (2010) 84(17):8903–12. doi: 10.1128/JVI.00851-10 PMC2919011 20573808

[6] Sun B Skjæveland I Svingerud T Zou J Jørgensen J Robertsen Børre. Antiviral activity of salmonid gamma interferon against infectious pancreatic necrosis virus and salmonid alphavirus and its dependency on type I interferon. J Virol (2011) 85(17):9188–98. doi: 10.1128/JVI.00319-11 PMC3165780 21697489

[7] Dehler CE Lester K Della Pelle G Jouneau L Houel A Collins C. Viral resistance and IFN signaling in STAT2 knockout fish cells. J Immunol (Baltimore Md. 1950) (2019) 203:465–75. doi: 10.4049/jimmunol.1801376 PMC6612602 31142600

[8] Abbas AK Lichtman AH Pillai S. Basic immunology. functions and disorders of the immune system. 6th edition. Baker DL Baker A, editors. Philadelphia: Elsevier (Medical Textbooks Immunology (2020).

[9] Chang M-X Zou J Nie P Huang B Yu Z Collet B. Intracellular interferons in fish: a unique means to combat viral infection. PloS Pathog (2013) 9(11):e1003736. doi: 10.1371/journal.ppat.1003736 24244163 PMC3828176

[10] Robertsen Børre. The role of type I interferons in innate and adaptive immunity against viruses in Atlantic salmon. Dev Comp Immunol (2018) 80:41–52. doi: 10.1016/j.dci.2017.02.005 28196779

[11] Chen SN Zou PF Nie P. Retinoic acid-inducible gene I (RIG-i)-like receptors (RLRs) in fish: current knowledge and future perspectives. Immunology (2017) 151(1):16–25. doi: 10.1111/imm.12714 28109007 PMC5382327

[12] Khan I Maldonado E Silva L Almeida D Johnson WE O’Brien SJ. The vertebrate TLR supergene family evolved dynamically by gene Gain/Loss and positive selection revealing a host–pathogen arms race in birds. Diversity (2019) 11(8):131. doi: 10.3390/d11080131

[13] Liao Z Su J. Progresses on three pattern recognition receptor families (TLRs, RLRs and NLRs) in teleost. Dev Comp Immunol (2021), 122:104131. doi: 10.1016/j.dci.2021.104131 34022258

[14] Seth RB Sun L Ea C-K Chen ZJ. Identification and characterization of *MAVS*, a mitochondrial antiviral signaling protein that activates NF-kappaB and IRF 3. Cell (2005) 122(5):669–82. doi: 10.1016/j.cell.2005.08.012 16125763

[15] Lauksund S Svingerud T Bergan V Robertsen Børre. Atlantic Salmon IPS-1 mediates induction of *IFNa*1 and activation of NF-kappaB and localizes to mitochondria. Dev Comp Immunol (2009) 33(11):1196–204. doi: 10.1016/j.dci.2009.06.012 19576240

[16] Dahle MK Jørgensen JB. Antiviral defense in salmonids - mission made possible? Fish shellfish Immunol (2019) 87:421–37. doi: 10.1016/j.fsi.2019.01.043 30708056

[17] Collet B Munro ES Gahlawat S Acosta F Garcia J Roemelt C. Infectious pancreatic necrosis virus suppresses type I interferon signalling in rainbow trout gonad cell line but not in Atlantic salmon macrophages. Fish shellfish Immunol (2007) 22(1-2):44–56. doi: 10.1016/j.fsi.2006.03.011 16713304

[18] Lauksund S Greiner-Tollersrud L Chang C-J Robertsen Børre. Infectious pancreatic necrosis virus proteins VP2, VP3, VP4 and VP5 antagonize *IFNa*1 promoter activation while VP1 induces *IFNa*1. Virus Res (2015) 196:113–21. doi: 10.1016/j.virusres.2014.11.018 PMC7114410 25445351

[19] Skotheim SA. Co-Infection with Norwegian salmonid alphavirus (NSAV) and infectious pancreatic necrosis virus (IPNV) in Chinook salmon embryo cells (CHSE-214). (2009) Bergen: The University of Bergen. Available at: https://bora.uib.no/bora-xmlui/handle/1956/3564.

[20] Gahlawat SK Ellis AE Collet B. Expression of interferon and interferon–induced genes in Atlantic salmon salmo salar cell lines SHK-1 and TO following infection with salmon AlphaVirus SAV. Fish shellfish Immunol (2009) 26(4):672–5. doi: 10.1016/j.fsi.2009.02.021 19264132

[21] Bela-Ong DB Greiner-Tollersrud L van der Wal WA Jensen I Seternes OM Jørgensen JB. Infection and microbial molecular motifs modulate transcription of the interferon-inducible gene ifit5 in a teleost fish. Dev Comp Immunol (2020) 111:103746. doi: 10.1016/j.dci.2020.103746 32445651

[22] Munir D Munro ES Secombes CJ Dooley H. Atlantic Salmon kidney (ASK) cells are an effective model to characterise interferon (IFN) and IFN-induced gene expression following salmonid alphavirus infection. Fish shellfish Immunol (2020) 106:792–5. doi: 10.1016/j.fsi.2020.08.043 32871248

[23] Le Cong RFA Cox D Lin S Barretto R Habib N Hsu PR. Multiplex genome engineering using CRISPR/Cas systems. Sci (New York N.Y.) (2013) 339(6121):819–23. doi: 10.1126/science.1231143 PMC3795411 23287718

[24] Hsu PD Lander ES Zhang F. Development and applications of CRISPR-Cas9 for genome engineering. Cell (2014) 157(6):1262–78. doi: 10.1016/j.cell.2014.05.010 PMC4343198 24906146

[25] Edvardsen RB Leininger S Kleppe L Skaftnesmo KO Wargelius A. Targeted mutagenesis in Atlantic salmon (Salmo salar l.) using the CRISPR/Cas9 system induces complete knockout individuals in the F0 generation. PloS One (2014) 9(9):e108622. doi: 10.1371/journal.pone.0108622 25254960 PMC4177897

[26] Dehler CE Boudinot P Martin SAM Collet B. Development of an efficient genome editing method by CRISPR/Cas9 in a fish cell line. Mar Biotechnol (New York N.Y.) (2016) 18(4):449–52. doi: 10.1007/s10126-016-9708-6 PMC5007268 27236514

[27] Gratacap RL Jin YeH Mantsopoulou M Houston RD. Efficient genome editing in multiple salmonid cell lines using ribonucleoprotein complexes. In Mar Biotechnol (New York N.Y.) (2020) 22:717–24. doi: 10.1007/s10126-020-09995-y PMC7520412 32946000

[28] Gratacap RL Regan T Dehler CE Martin SAM Boudinot P Collet B. Efficient CRISPR/Cas9 genome editing in a salmonid fish cell line using a lentivirus delivery system. BMC Biotechnol (2020) 20(1):35. doi: 10.1186/s12896-020-00626-x 32576161 PMC7310381

[29] Monjo AL Poynter SJ DeWitte-Orr SJ. CHSE-214: a model for studying extracellular dsRNA sensing *in vitro* . Fish shellfish Immunol (2017) 68:266–71. doi: 10.1016/j.fsi.2017.07.025 28705724

[30] Reed LJ Muench H. A simple method of estimating fifty per cent Endpoints12. Am J Epidemiol (1938) 27(3):493–7. doi: 10.1093/oxfordjournals.aje.a118408

[31] Jenberie S Peñaranda MaMD Thim HL Styrvold MB Strandskog G Jørgensen JB. Salmonid alphavirus subtype 3 induces prolonged local b cell responses in Atlantic salmon (Salmo salar) after intraperitoneal infection. Front Immunol (2020) 11:1682. doi: 10.3389/fimmu.2020.01682 33013821 PMC7511533

[32] Brinkman EK Chen T Amendola M van Steensel B. Easy quantitative assessment of genome editing by sequence trace decomposition. Nucleic Acids Res (2014) 42(22):e168. doi: 10.1093/nar/gku936 25300484 PMC4267669

[33] Iliev DB Thim H Lagos L Olsen R Jørgensen JB. Homing of antigen-presenting cells in head kidney and spleen - salmon head kidney hosts diverse APC types. Front Immunol (2013) 4:137. doi: 10.3389/fimmu.2013.00137 23761795 PMC3674399

[34] Jenberie S Thim HL Sunyer JO Skjødt K Jensen I Jørgensen JB. Profiling Atlantic salmon b cell populations: CpG-mediated TLR-ligation enhances IgM secretion and modulates immune gene expression. In Sci Rep (2018) 8(1):3565. doi: 10.1038/s41598-018-21895-9 PMC5824956 29476080

[35] Robertsen Børre Greiner-Tollersrud L Jørgensen LG. Analysis of the Atlantic salmon genome reveals a cluster of mx genes that respond more strongly to IFN gamma than to type I IFN. Dev Comp Immunol (2019) 90:80–9. doi: 10.1016/j.dci.2018.09.004 30195710

[36] Sobhkhez M Joensen LL Tollersrud LG Strandskog G Thim HL Jørgensen JB. A conserved inhibitory role of suppressor of cytokine signaling 1 (SOCS1) in salmon antiviral immunity. Dev Comp Immunol (2017) 67:66–76. doi: 10.1016/j.dci.2016.11.001 27818171

[37] Schmittgen TD Livak KJ. Analyzing real-time PCR data by the comparative C(T) method. Nat Protoc (2008) 3(6):1101–8. doi: 10.1038/nprot.2008.73 18546601

[38] Li C Greiner-Tollersrud L Robertsen Børre. Infectious salmon anemia virus segment 7 ORF1 and segment 8 ORF2 proteins inhibit IRF mediated activation of the Atlantic salmon *IFNa*1 promoter. Fish Shellfish Immunol (2016) 52:258–62. doi: 10.1016/j.fsi.2016.03.038 27012395

[39] Strandskog G Villoing Stéphane Iliev DB Thim HL Christie KE Jørgensen JB. Formulations combining CpG containing oliogonucleotides and poly I:C enhance the magnitude of immune responses and protection against pancreas disease in Atlantic salmon. Dev Comp Immunol (2011) 35(11):1116–27. doi: 10.1016/j.dci.2011.03.016 21527278

[40] Bergan V Kileng Øyvind Sun B Robertsen Børre. Regulation and function of interferon regulatory factors of Atlantic salmon. Mol Immunol (2010) 47(11-12):2005–14. doi: 10.1016/j.molimm.2010.04.015 20494444

[41] Westermann L Li Y Göcmen B Niedermoser M Rhein K Jahn J. Wildtype heterogeneity contributes to clonal variability in genome edited cells. Sci Rep (2022) 12(1):18211. doi: 10.1038/s41598-022-22885-8 36307508 PMC9616811

[42] Au WC Moore PA LaFleur DW Tombal B Pitha PM. Characterization of the interferon regulatory factor-7 and its potential role in the transcription activation of interferon a genes. J Biol Chem (1998) 273(44):29210–7. doi: 10.1074/jbc.273.44.29210 9786932

[43] Sharma S tenOever BR Grandvaux N Zhou G-P Lin R Hiscott J. Triggering the interferon antiviral response through an IKK-related pathway. Sci (New York N.Y.) (2003) 300(5622):1148–51. doi: 10.1126/science.1081315 12702806

[44] Dalskov L Narita R Andersen LL Jensen N Assil S Kristensen KH. Characterization of distinct molecular interactions responsible for IRF3 and IRF7 phosphorylation and subsequent dimerization. Nucleic Acids Res (2020) 48(20):11421–33. doi: 10.1093/nar/gkaa873 PMC7672473 33205822

[45] Servant MJ Tenoever B Lin R. Overlapping and distinct mechanisms regulating IRF-3 and IRF-7 function. J Interferon Cytokine Res (2002) 22(1):49–58. doi: 10.1089/107999002753452656 11846975

[46] Ivashkiv LB Donlin LT. Regulation of type I interferon responses. Nat Rev Immunol (2014) 14(1):36–49. doi: 10.1038/nri3581 24362405 PMC4084561

[47] Loo Y-M Fornek J Crochet N Bajwa G Perwitasari O Martinez-Sobrido L. Distinct RIG-I and MDA5 signaling by RNA viruses in innate immunity. J Virol (2008) 82(1):335–45. doi: 10.1128/JVI.01080-07 PMC2224404 17942531

[48] Perry ST Prestwood TR Lada SM Benedict CA Shresta S. Cardif-mediated signaling controls the initial innate response to dengue virus *in vivo* . J Virol (2009) 83(16):8276–81. doi: 10.1128/JVI.00365-09 PMC2715757 19494017

[49] Skjesol A Skjæveland I Elnæs M Timmerhaus G Fredriksen BørgeN Jørgensen SM. IPNV with high and low virulence: host immune responses and viral mutations during infection. Virol J (2011) 8:396. doi: 10.1186/1743-422X-8-396 21827718 PMC3169513

[50] de Sena J Rio GJ. Partial purification and characterization of RTG-2 fish cell interferon. Infection Immun (1975) 11(4):815–22. doi: 10.1128/iai.11.4.815-822.1975 PMC415140 235493

[51] Reyes-Cerpa Sebastián Reyes-López FE Toro-Ascuy D Ibañez J Maisey K Sandino AMaría. IPNV modulation of pro and anti-inflammatory cytokine expression in Atlantic salmon might help the establishment of infection and persistence. Fish shellfish Immunol (2012) 32(2):291–300. doi: 10.1016/j.fsi.2011.11.018 22142704

