# Supplementary material for: CRISPR-Cas– induced IRF3 and MAVS knockouts in a salmonid cell line disrupt PRR signaling and affect viral replication
Source: Front Immunol. 2023 Jul 17;14:1214912. doi: 10.3389/fimmu.2023.1214912 (PMC10425769; doi:10.3389/fimmu.2023.1214912)
Supplement: Supplementary file 1 [file DataSheet_1.pdf]

Supplementary Material

CRISPR-Cas induced IRF3 and MAVS knockouts in a salmonid cell line disrupt PRR signaling and affect viral replication.

Yorick A. van der Wal, Henriette Nordli, Allan Akandwanaho, Linn Greiner-Tollersrud, Jaap Kool, Jorunn B. Jørgensen*

*** Correspondence:** Jorunn B. Jørgensen: jorunn.jorgensen@uit.no

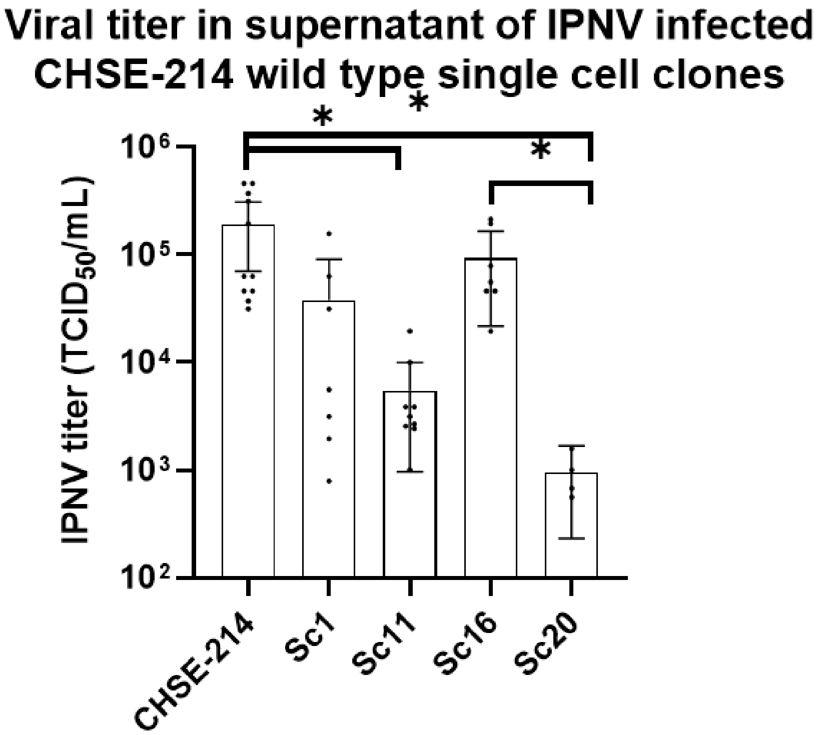

Supplementary figure 1: IPNV growth on wild type single cell clones of CHSE-214 cells. IPNV titers in supernatants of infected wild type single cell clones 2 days post infection. Values of duplicates or triplicates from two (Sc20), three (Sc1, Sc16), or five (CHSE-214, Sc11) experiments visualized as dots, and error bars indicate 95% confidence interval. (*) Statistically significant differences between wild type clones and/or original CHSE-214 pool.

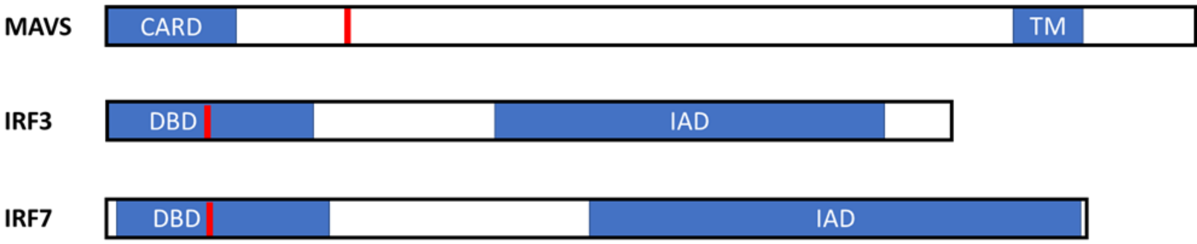

Supplementary Figure 2: Schematic locations of domains and introduced mutations in MAVS, IRF3, and IRF7 edited CHSE-214 cells. Blue boxes indicate important functional domains: DNA-binding domain (DBD), caspase activation recruitment domain (CARD), IRF association domain (IAD), and transmembrane domain (TM). The red line indicates the location of the induced mutation in tested single cell clones.

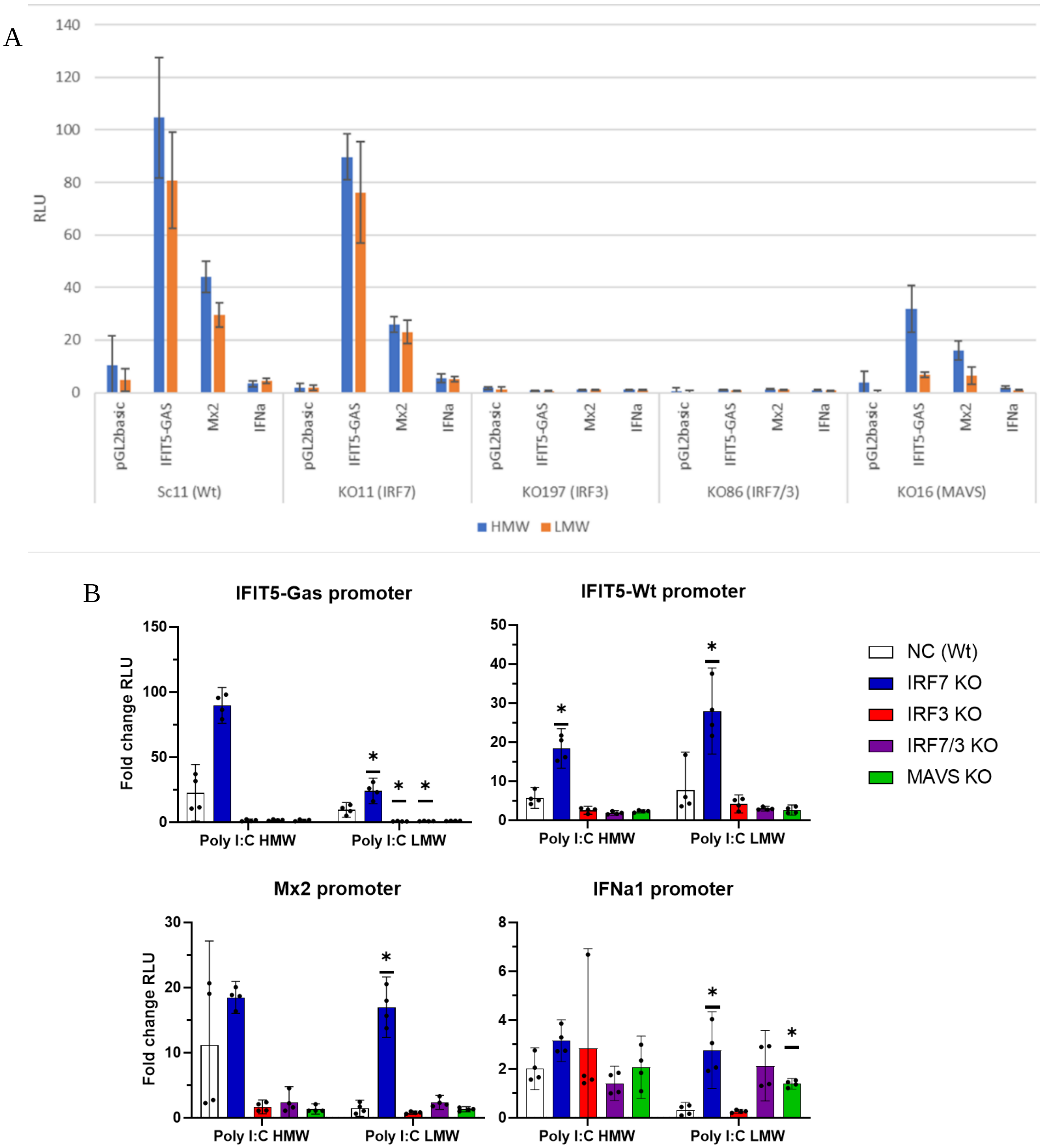

Supplementary figure 3: Promoter activation in IRF7, IRF3, and MAVS KO CHSE-214 cells 48 hours after HMW or LWM poly I:C transfection. A: Alternative representation of data from figure 2. The graph shows the means of the RLU normalized against co-transfected Renilla plasmid for non-stimulated, HMW poly I:C, and LMW poly I:C transfected cells. B: Data from a second promoter activation experiment. The graphs show the fold change of RLU (normalized against co-transfected Renilla plasmid) compared to non poly I:C transfected controls. Values of the quadruplicates visualized as dots, and error bars indicate 95% confidence interval. (*) Statistically significant different from the wild type NC.

Supplementary table 1: CRISPR/Cas induced mutations and premature stop codons in investigated KO single cell clones.

Single cell clone	Gene/ allele	Indel	Genome sequence at mutation site	Amino acids until (premature) stop codon
Sc11	IRF7	Wt	CACAATTCTAGGAAG<br>GATTGCTCCGAGGA	MQSCKPQFADWLIEQVRTEQYTGLFFIDNNKFRVPWKHNSRKDCSEDDRKI<br>FRAWAVVSGKINEHPTDKAKWKTNFRSALNSLCRRFKMVEDHSKDSNDPH<br>KVYLVINEYNYENPHIEEITLENYGLDCALIPTENTPPGMEHDILNFSNLTLNPL<br>DLNQHTENSIPVHTHHSVPPVLVQQPYYQVNPDALLNLPAAHSSLWDLEITI<br>SYRGSEMRKTQVSGPRVQLHYQCNALEPNTQPLCFPSTDGLPDHKQ
	IRF3	Wt	GCTGCGCGAACAGAT<br>AAATAGTGCCGGT	MSQSKPLLIPWLREQINSGRYPGVTWTNQERTEFCIPWKHALRQDSCSDDV<br>LIFKAWAEVSNGRVQGDHSIWKRNFRSALRAKGFKMLLDNKNDAANPNKL<br>FQWPDEAPTGGSQHPEHDLYQDPSPLQESHGLPCFNDLYLAAEETVYTAEG<br>ISTTINQDILQKCLQGLNIEQTEAIQGYEVPVEELQYPMEGTIGGHVLLGQQQ<br>YPVVMEDAVGGAVLPSQPVYPMDGAVGGSHEQQVVEQLTNELSRTMVG<br>ENFKTHFRVSVYYRGVKMPEQLVENEAGFRLVYSSVSTRPELTQPLLDPDSG<br>LNLVSLPSPPPVQDETQAKLTQDILALLGEGLEVGASGSIIYGLRKGEIKAFWS<br>LDKFDNSRRPQGVSKCPEPLYQAKDFYG
	MAVS	Wt	sgRNA (2) site:<br>CAGCCCTGGAGCTGT<br>TGGAGCATCAGGAT<br><br>sgRNA (3) site:<br>CAATTCCTTCTACCAG<br>CTCTGAGGGGTCGC	MSSFTREKLSLHLRRRMGVFVSRVKATELMANLPCLTPSDKEEIQAKKDFSG<br>NYAAMQLLLDYVQKRMNWPEELMSALELLEHQDLADELRAEWNKHNQN<br>NPYPPSPAATTTVRTHVHPIPSTSSEGSPCSLVLPGQPAPPEVAAPPEASLPPE<br>VAPEVLPPPVVAAQPEAPPRSVPKAPMAGSSSKHAPKAAVSPEIASEAAPSP<br>VAAPQAEPQAAPLSPVSVEEPTVISEPPASSQPGSIETVSLEDNLCHSDAPTQ<br>MALSETTPTLSGSHLIPVVSEITPTLPVSHLALSQTESTPTPAALATFQSPERRP<br>VQDTSPHTVKVPTFYQEAVDSDPTQVTEDEQHTEPSQSQHFATAPADTSM<br>NEDDVNFSKPEVLRSEVMDSQPYSGDSTRLQRRMEFLRK*
KO11	IRF7	+1<br>T	CACAATTCTAGGAAG<br>GATTGCTTCCGAGGA	MQSCKPQFADWLIEQVRTEQYTGLFFIDNNKFRVPWKHNSRKDCFRGRP*
KO197	IRF3<br>allele 1	-2<br>GT	GCTGCGCGAACAGAT<br>AAATA--GGCCGGT	MSQSKPLLIPWLREQINRPVSRGYLDQSGANRVLHPLETCFEAGFLQR*
	IRF3<br>allele 2	+1<br>T	GCTGCGCGAACAGAT<br>AAATAGTTGGCCGGT	MSQSKPLLIPWLREQINSWPVSRGYLDQSGANRVLHPLETCFEAGFLQR*
KO86	IRF7	+1<br>T	CACAATTCTAGGAAG<br>GATTGCTTCCGAGGA	MQSCKPQFADWLIEQVRTEQYTGLFFIDNNKFRVPWKHNSRKDCFRGRP*
	IRF3	-2<br>TG	GCTGCGCGAACAGAT<br>AAATAG--GCCGGT	MSQSKPLLIPWLREQINRPVSRGYLDQSGANRVLHPLETCFEAGFLQR*
KO16	MAVS<br>allele 1	-2<br>CT	CAATTCCTTCTACCAG<br>CT--GAGGGGTCGC	MSSFTREKLSLHLRRRMGVFVSRVKATELMANLPCLTPSDKEEIQAKKDFSG<br>NYAAMQLLLDYVQKRMNWPEELMSALELLEHQDLADELRAEWNKHNQN<br>NPYPPSPAATTTVRTHVHPIPSTS*
	MAVS<br>allele 2	+1<br>T	CAATTCCTTCTACCAG<br>CTCTTGAGGGGTCGC	MSSFTREKLSLHLRRRMGVFVSRVKATELMANLPCLTPSDKEEIQAKKDFSG<br>NYAAMQLLLDYVQKRMNWPEELMSALELLEHQDLADELRAEWNKHNQN<br>NPYPPSPAATTTVRTHVHPIPSTSS*

T: insertion, - : deletion, * : premature stopcodon.

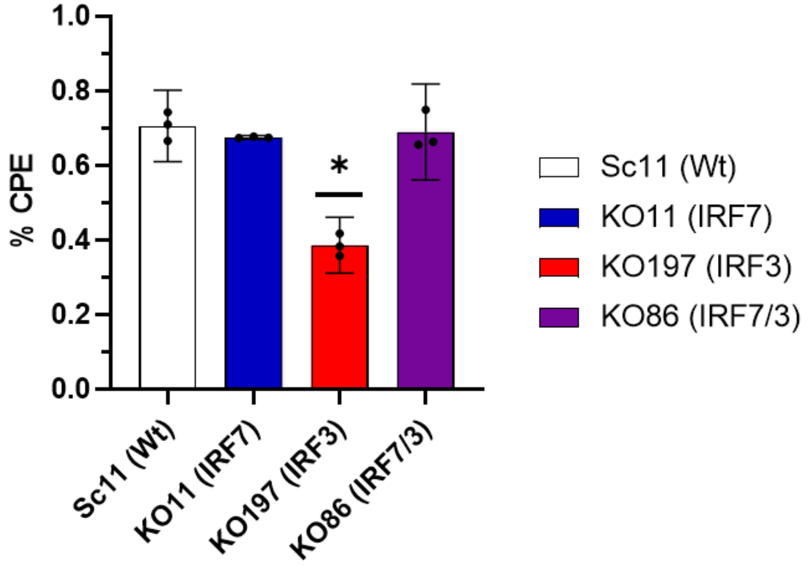

Supplementary figure 4: CPE on IPNV infected IRF7 and IRF3 KO CHSE-214 cells 2 days post infection. Crystal violet staining of IPNV infected cell monolayers was measured at OD590 as indication of confluence. % CPE was calculated as follows: 1 – OD(infected) / OD(non-infected). Values of single wells from three experiments visualized as dots, and error bars indicate 95% confidence interval. (*) Statistically significant different from the wild type Sc11.

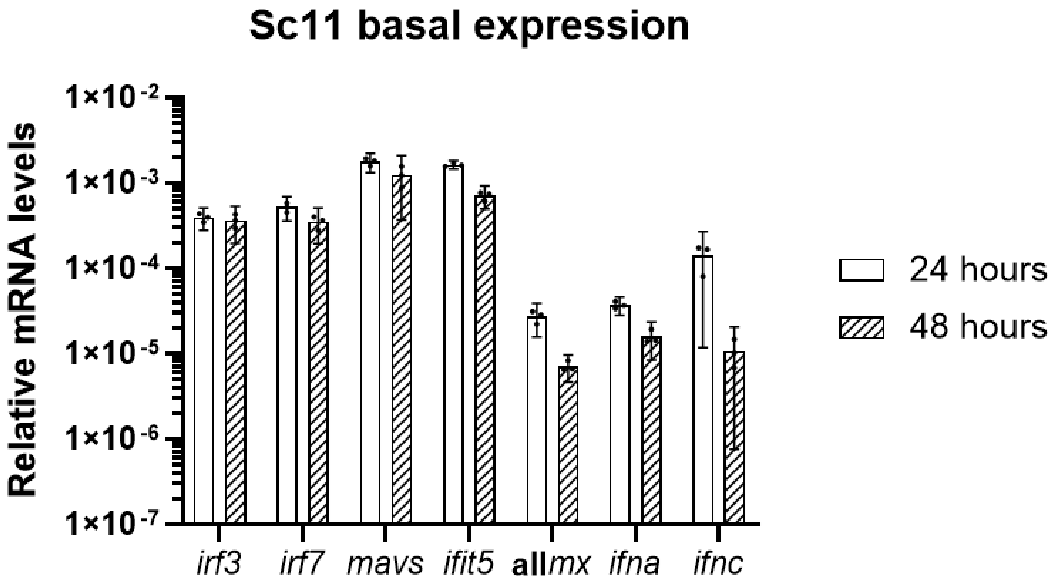

Supplementary figure 5: Basal expression of ifit5, allmx, ifna, ifnc, irf3, irf7, and mavs in wildtype CHSE-214 cells 24 and 48 hours after mock stimulation. The graphs show the mRNA levels relative to elf2a. Values of the triplicates visualized as dots, and error bars indicate 95% confidence interval.

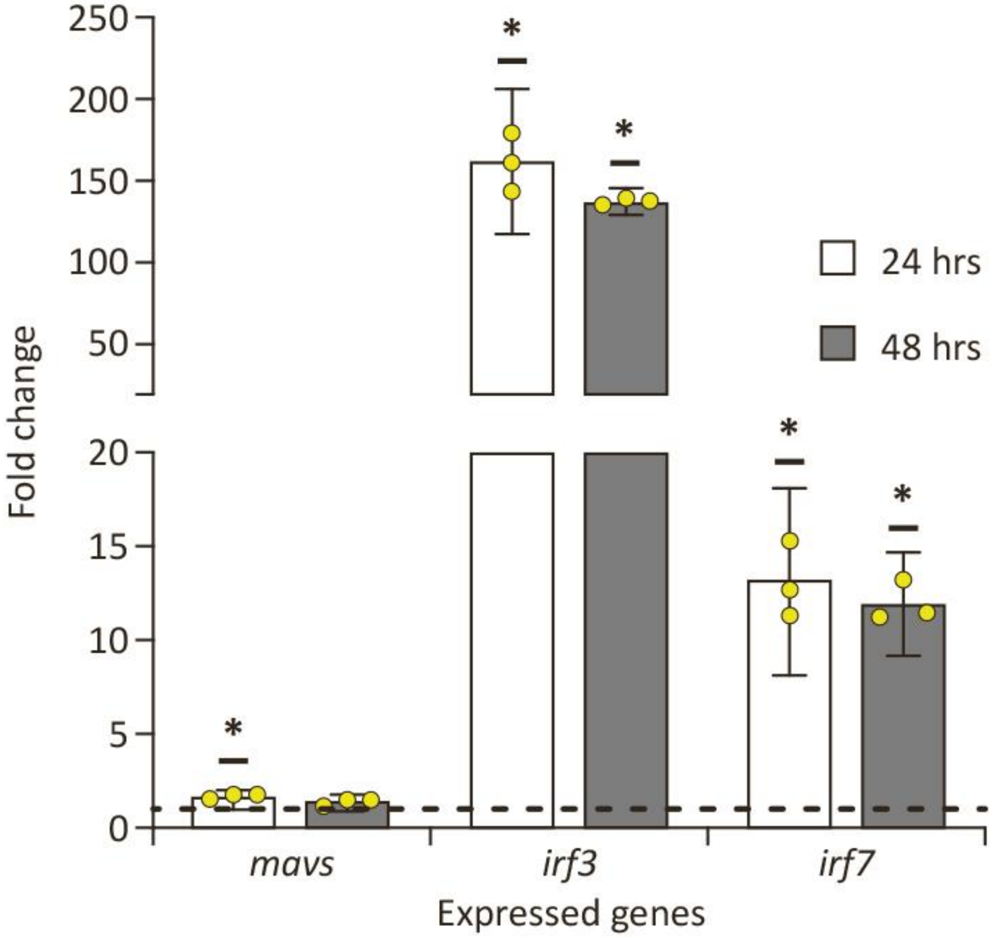

Supplementary figure 6: Expression of *mavs*, *irf3*, and *irf7* in NC 24 and 48 hours after HMW poly I:C transfection. The graphs show the fold change of expression compared to non poly I:C transfected controls and normalized against *elf2a*. Values of the triplicates visualized as dots, and error bars indicate 95% confidence interval. (*) Statistically significant difference (*) from mean 1 (no change, dotted line) based one-sample t-test.
