# Supplementary material for: CRISPR-Cas– induced IRF3 and MAVS knockouts in a salmonid cell line disrupt PRR signaling and affect viral replication
Source: Front Immunol. 2023 Jul 17;14:1214912. doi: 10.3389/fimmu.2023.1214912 (PMC10425769; doi:10.3389/fimmu.2023.1214912)
Supplement: Supplementary file 2 [file DataSheet_2.pdf]

Supplementary material – Duplicate genes

The genes for IRF3, IRF7, and MAVS from rainbow trout and Atlantic salmon were aligned per gene, and sequencing primers were designed in homologous regions. These primers enabled sequencing of the respective genes from the chinook salmon genome as isolated from CHSE-214 cells, and a blast against the chinook salmon genome in ncbi confirmed the genetic locations and sequences.

Table: Locations and predictions of genomic locations

Gene	Sequence	Location	Annotation ncbi	Sequence ID
IRF3	Oncorhynchus tshawytscha isolate Ot180627B linkage group LG24, Otsh_v2.0, whole genome shotgun sequence	5299671 - 5302798	Predicted IRF3	NC_056452.1
IRF7	Oncorhynchus tshawytscha isolate Ot180627B linkage group LG06, Otsh_v2.0	36074493 - 36084407	Predicted IRF7-like	NC_056434.1
MAVS	Oncorhynchus tshawytscha isolate Ot180627B linkage group LG11, Otsh_v2.0	55045023 - 55053498	Predicted Proline-rich protein 36	NC_056439.1

We identified possible duplicate genes by a blast of the coding sequences in the ncbi nucleotide collection. IRF3 only showed homology with IRF3 transcript variants. IRF7 had 80% homology with a predicted mRNA for IRF8, along with being identical to predicted IRF7 mRNA. The IRF8 mRNA was aligned in Clustal omega with IRF7 and IRF8 from *Homo sapiens*, *Danio rerio*, and *Oncorhynchus mykiss* and clustered with IRF8, so is probably the IRF8 mRNA and not a IRF7 duplicate.

Figure: Phylogenetic tree of IRF7 and IRF8 coding sequences

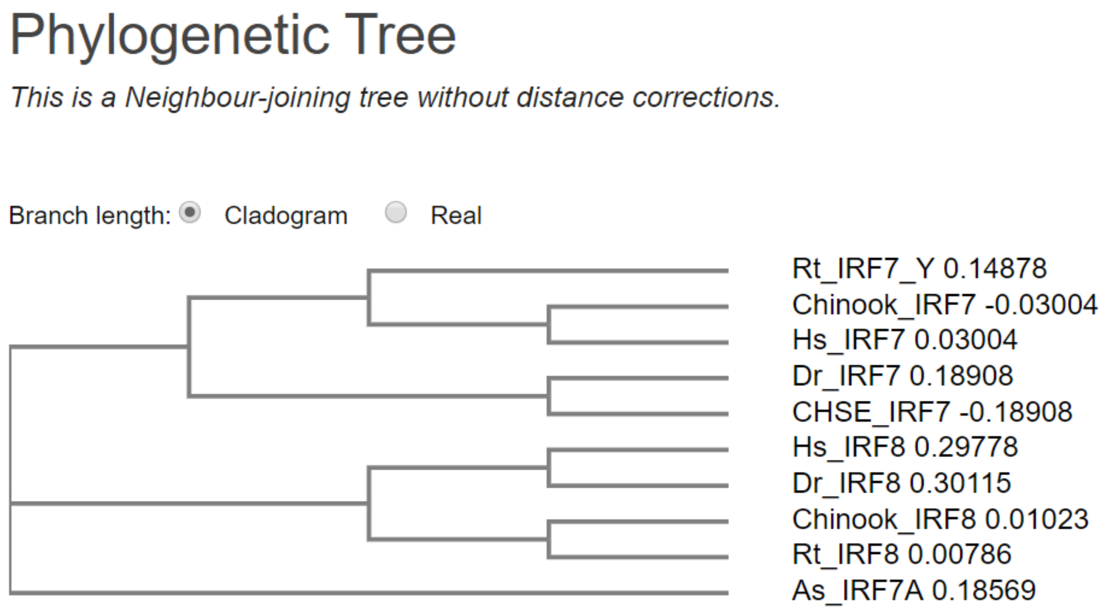

MAVS corresponded to a predicted proline-rich protein 36 mRNA and had 66% homology with the predicted MAVS mRNA. The gene we found while searching for MAVS was also predicted to be PRP36, so we set out to check these predictions. An alignment with the CDSs of *Homo sapiens*, *Mus musculus*, *Danio rerio*, and *Oncorhynchus mykiss* MAVS and PRP36 showed grouping of both mRNAs with MAVS, with the predicted PRP36 mRNA being closest to the other salmonid MAVS sequences.

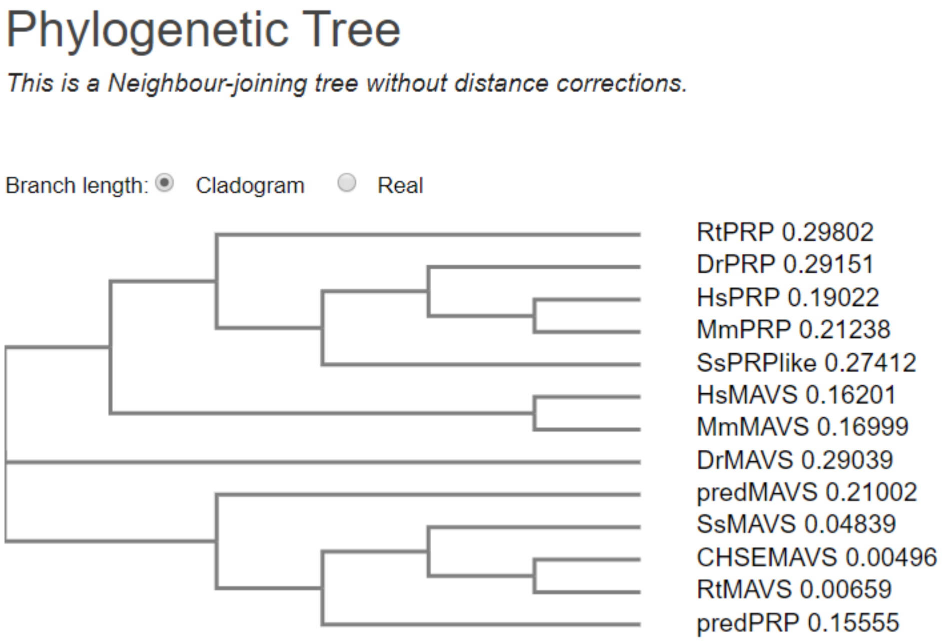

Figure: Phylogenetic tree of MAVS and PRP36 coding sequences

The predicted PRP36 in chinook salmon thus seems to be actually MAVS and will now be called ChMAVSa, while the mRNA predicted to be MAVS could be a duplicate gene and will be called ChMAVSb. These conclusions are corroborated by domain predictions. ChMAVSa has a very clear CARD domain, while in ChMAVSb only the death domain superfamily was recognized. Both proteins have a transmembrane domain at the C terminus.

We blasted both mRNA sequences against the chinook salmon, rainbow trout, and Atlantic salmon ncbi RefSeq Genome Databases to find the corresponding genes and to investigate the gene synteny.

Table: Genomic locations of genes corresponding to ChMAVSa and ChMAVSb

Name used here	Species	Predicted in ncbi	Location	Accession no. ncbi
**ChMAVSa**				XM_042330395
ChMAVSa	Chinook salmon	proline-rich protein 36	LG11 - end	LOC112236223
RtMAVSa	Rainbow trout	MAVS	Chr. 19 - end	100499614 (mavs)
AsIPS-1	Atlantic salmon	IPS-1 (MAVS)	Chr. 01 - start	LOC100316613
**ChMAVSb**				XM_042326218
ChMAVSb	Chinook salmon	MAVS	LG08 - start	112237596 (mavs)
RtMAVSb	Rainbow trout	mRNA-nascent polypeptide-associated complex subunit alpha, muscle-specific form	Chr. 25 - end	LOC110504282
AsMAVSb	Atlantic salmon	MAVS-like / mucin-6-like	Chr. 9 - 1/3	LOC106611470

Synteny for ChMAVSa compares very well between all three species, indicating that ChMAVSa is actually MAVS as described for Atlantic salmon (Lauksund 2009, Biacchesi 2009). The synteny of ChMAVSb compares well with the RtMAVSb gene (predicted nascent polypeptide-associated complex subunit alpha, muscle-specific form), and to a lesser extend to the AsMAVSb gene (predicted MAVS-like or mucin-6-like). Interestingly, AsMAVSb is predicted to be MAVS-like, has two genes in the vicinity that are near the actual ChMAVSa, and is on a location of chromosome 9 that was duplicated from the start of chromosome 1, where the AsIPS-1 (MAVS) gene is situated. This all suggests that this gene might be a duplicate of MAVS. The ChMAVSb and AsMAVSb proteins both contain a death domain superfamily domain that indicates a possible CARD domain at the N terminus and a transmembrane domain at the C terminus, suggesting a function similar to MAVS. The RtMAVSb protein lacks these domains, however, and will not have a MAVS-like function.

sav1 >	atl1 <	cdkl1 >	l2hgdh >	sos2 >	cn1h1 <	pacs2 >	dcaf5 >	map3k9 >	pcnx1 >	fbln5 >	plek2 >	smor1 <	paplna <	exd2 <	wdr32 >	manba >	nat8 >	si:dky-33c <	ChMAVSa <	rab11fip5a >	cfd >
jade1 >	cfd >	rab11fip5a <	RtMAVSa <	si:dky-33c12.3 <	nat8 >	manba >	wdr32 >	paplna <	smor1 <	plek2 >	pcnx1 <	med6 >	map3k9 <	cn1h1 <	sos2 >	l2hgdh >	atl1 <	sav1 >			
jade1 >	manba >	AsIPS-1 >	exd2 >	smor1 >	pcnx >	fbln5 >	plek2 >	med6 >	map3k9 <	sav1 >	atl1 <	cdkl1 >	l2hgdh >	sos2 >	cn1h1 <						
asmt2 <	letm1 >	fgfr3 <	eif2ak3 >	ChMAVSb >	pank2 >	slc39a8 <	zmp:0000(<	marc1 <	capn3a <	uti1b <	golga5 <										
capn3a >	nenf >	marc1 >	zmp:00000(>	slc39a8 >	mrps26 >	pank2 <	RtMAVSb <	ap5s1 >	eif2ak3 <	fgfr3 >	letm1 <	asmt2 >									
nenf >	ap5s1 <	eif2ak3 >	pacs2 >	mrps26 >	AsMAVSb <	golga5 >	dcaf5 <	uti1b <													

Figure: Synteny of chinook salmon, rainbow trout, and Atlantic salmon genes corresponding to ChMAVSa and ChMAVSb

MAVS gene/duplicate
Strong synteny in the three species
Synteny in chinook salmon and rainbow trout
Synteny in Atlantic salmon and rainbow trout
Synteny in Atlantic salmon and chinook salmon
Present near Atlantic salmon MAVS duplicate and chinook salmon MAVS gene
Genes with largely conserved gene order

Legend
